# Phytotoxic Secondary Metabolites from Fungi

**DOI:** 10.3390/toxins13040261

**Published:** 2021-04-06

**Authors:** Dan Xu, Mengyao Xue, Zhen Shen, Xiaowei Jia, Xuwen Hou, Daowan Lai, Ligang Zhou

**Affiliations:** Department of Plant Pathology, College of Plant Protection, China Agricultural University, Beijing 100193, China; cauxudan@cau.edu.cn (D.X.); mengyaoxue@cau.edu.cn (M.X.); zhenshen@cau.edu.cn (Z.S.); xiaoweijia@cau.edu.cn (X.J.); xwhou@cau.edu.cn (X.H.); dwlai@cau.edu.cn (D.L.)

**Keywords:** fungi, plant pathogenic fungi, phytotoxic, herbicidal, phytotoxins, mycotoxins, mycoherbicides, secondary metabolites

## Abstract

Fungal phytotoxic secondary metabolites are poisonous substances to plants produced by fungi through naturally occurring biochemical reactions. These metabolites exhibit a high level of diversity in their properties, such as structures, phytotoxic activities, and modes of toxicity. They are mainly isolated from phytopathogenic fungal species in the genera of *Alternaria*, *Botrytis*, *Colletotrichum*, *Fusarium*, *Helminthosporium*, and *Phoma*. Phytotoxins are either host specific or non-host specific phytotoxins. Up to now, at least 545 fungal phytotoxic secondary metabolites, including 207 polyketides, 46 phenols and phenolic acids, 135 terpenoids, 146 nitrogen-containing metabolites, and 11 others, have been reported. Among them, aromatic polyketides and sesquiterpenoids are the main phytotoxic compounds. This review summarizes their chemical structures, sources, and phytotoxic activities. We also discuss their phytotoxic mechanisms and structure–activity relationships to lay the foundation for the future development and application of these promising metabolites as herbicides.

## 1. Introduction

Phytotoxic secondary metabolites from fungi (or called fungal phytotoxins) are toxic compounds to plants produced by fungi, especially by plant fungal pathogens responsible for serious diseases of agrarian and forest plants causing significant economical losses [1]. Fungal phytotoxins play an important role in the development of plant disease symptoms, inclduing leaf spots, wilting, chlorosis, necrosis, and growth inhibition and promotion [2,3]. Their chemical and biological characterizations as well as the structure–activity relations and modes of action can help us to deeply investigate plant-pathogen interactions.

Fungal phytotoxins are either host specific (HST) or non-host specific (NHST) toxins. Hosts specific phytotoxins (or called host-selective toxins) are active only towards plants that are hosts of the toxin-producing fungi, and are essential for pathogenicity [4]. Host specific toxins are mainly produced by plant pathogenic fungi of *Alternaria*, *Colletotrichum*, and *Helminthosporium* [5,6]. In some cases, host sensitivity is mediated by gene-for-gene interactions, and phytotoxin sensitivity is mandatory for disease development [7]. Contrarily, non-host specific phytotoxins (or called non-host-selective toxins) are primary determinants of host range and not essential for pathogenicity, although they may contribute to virulence. These phytotoxins have a broader range of activity, causing symptoms not only on hosts of the pathogenic fungi, but also on other plant species [8].

Many reviews related to the phytotoxins produced by fungi have been previously published. However, some reviews have been published for a few years [1,8,9,10,11]. Some discussed only the biochemical and physiological aspects of phytotoxins [12], and some others only proposed fungal phytotoxins as potential herbicides [11,13]. Additionally, other reviews only reported on the phytotoxins produced by one fungal genus [4,14,15,16], or one fungal species [17]. Some others were reported on the phytotoxins produced by fungi during interactions with one plant species or only one plant group [18]. Furthermore, with the expansion of research scope, more and more fungal phytotoxins with herbicidal potential have been recently revealed from non-phytopathogenic fungi such as plant endophytic fungi [19,20,21], animal endophytic fungi [22,23,24], soil-derived fungi [25,26], and marine-derived fungi [27,28].

Fungal phytotoxins belong to different classes such as polyketides, phenols and phenolic acids, terpenoids, nitrogen-containing metabolites based on their biosynthetic pathways and structural characters. To our knowledge, there are no detailed reviews about the phytotoxic secondary metabolites from all fungal species to be published. This review describes fungal phytotoxic metabolites, their structures, isolated fungi and phytotoxic activities. Furthermore, the probable roles played by fungal phytotoxins in the induction of plant disease symptoms, structure–activity relationships, phytotoxic mechanisms, as well as the potential applications in agriculture are also discussed.

## 2. Polyketides

Polyketides are an extremely important class of bioactive secondary metabolites. They are produced by repetitive Claisen condensations of an acyl-coenzyme A (CoA) starter with malonyl-CoA elongation units in a fashion reminiscent of fatty acid biosynthesis. The biosynthesis of polyketides from acyl-CoA thioesters is catalyzed by polyketide synthase (PKS), a multi-enzyme complex that is highly homologous to fatty acid synthase (FAS). The diverse structures of polyketides can be explained as being derived from poly-β-keto chains, formed by the coupling of acetic acid units via condensation reactions. Although sharing a similar synthetic process, PKSs can be classified into three types, namely type I PKS, type II PKS, and type III PKS. Type I PKSs are multifunctional peptides containing linearly arranged and covalently fused domains. The type I PKSs can be further classified into iterative type I PKSs (iPKSs) and modular type I PKSs (mPKSs). Type II PKSs are multi-enzyme complexes composed of monofunctionall proteins. Type III PKSs are simple homodimers that use CoA rather than acyl carrier protein (ACP) as an anchor for chain extension. In addition, both type II and type III PKSs are iterative [29]. Most fungal phytotoxic metabolites belong to polyketides. They are mainly divided into aromatic and aliphatic polyketides.

### 2.1. Aromatic Polyketides

Aromatic polyketides are characterized by their polycyclic aromatic structures. The biosynthesis of aromatic polyketides is usually accomplished by the type II polyketide synthases (PKSs), which produce highly diverse polyketide chains by sequential condensation of the starter units with extender units, followed by reduction, cyclization, aromatization and tailoring reactions [29]. Many fungal phytotoxic polyketides belong to aromatic polyketides that mainly include benzopyrones, dibenzopyrones, benzophenones, naphthopyrones, azaphilones, naphthalenes, anthraquinones, perylenequinonoids, and aromatic macrolides.

#### 2.1.1. Benzopyrones

**Figure 1:**
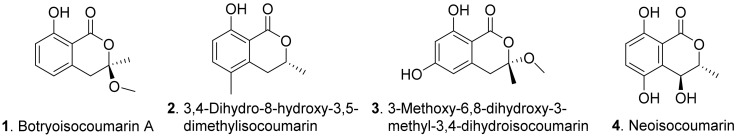

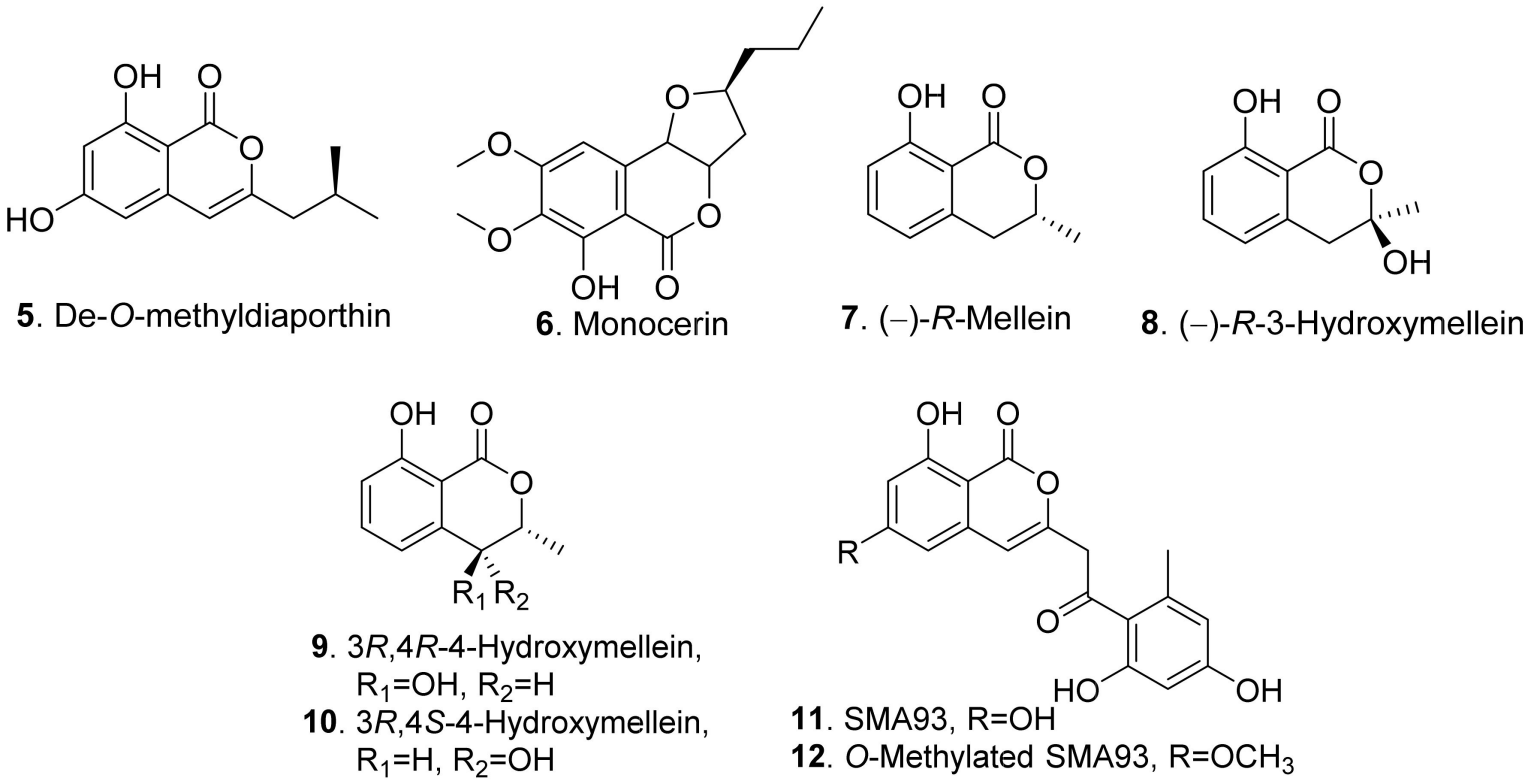
Structures of the phytotoxic benzo-α-pyrones isolated from fungi.

Phytotoxic benzopyrones from fungi mainly include benzo-α-pyrones and benzo-γ-pyrones. Benzo-α-pyrones are also called isocoumarin derivatives. The structures of fungal phytotoxic benzo-α-pyrones are shown in Figure 1.

Botryoisocoumarinn A (**1**) and neoisocoumarin (**4**) were isolated from *Neufusicoccum batangarum*, the causal agent of the scabby canker of cactus pear (*Opuntia ficus-indica*). Both metabolites induced a necrotic lesions around the inoculation points in both host (cactus pear) and non-host (tomato) plants [30].

Then, 3,4-dihydro-8-hydroxy-3,5-dimethyl isocoumarin (also called 5-methylmellein, **2**) was isolated from the culture broth of *Diaporthe eres*, the leaf pathogen of *Hedera helix*. This compound caused necrotic lesions on the leaves of *H. helix*. It inhibited seed germination and seedling growth of lettuce (*Lactuca sativa*) and bentgrass (*Agrostis stolonifera*). It was much more phytotoxic to bentgrass than to lettuce [31].

In addition, 3-methoxy-6,8-dihydroxy-3-methyl-3,4-dihydroisocoumarin (**3**) was isolated from *Pyricularia grisea*, the pathogen of buffelgrass (*Cenchrus ciliaris*). This metabolite showed a significant stimulating effect on the radical elongation of buffelgrass by seedling growth assay [32].

De-*O*-methyldiaporthin (**5**) was isolated from the liquid culture of *Drechslera siccans* which was the pathogen of oat (*Aoena sativa*), perennial ryegrass (*Lolium perenne*), and Italian ryegrass (*Lolium multiflorum*). Leaf necrosis were observed on corn (*Zea mays*), crabgrass (*Digitaria ischaemum*), and soybean (*Glycine max*) [33].

Monocerin (**6**) was isolated from *Eserohilum turcicum* (syn. *Drechslera turcica*), the leaf pathogen of the noxious weed Johnson grass (*Sorghum halepense*). This metabolite possessed non-specific phytotoxic activity to inhibit root and shoot growth of Johnson grass and cucumber seedlings [34].

(−)-*R*-Mellein (**7**) was isolated from the culture broth of *Phoma tracheiphila*, the pathogen causing the citrus disease known as mal secco. (−)-*R*-Mellein (**7**) induced symptoms of phytotoxicity in tomato cuttings at 100 μg/mL [35]. (−)-*R*-Mellein (**7**) was also isolated from *Sardiniella urbana*, the emerging pathogen on European hackberry [36], and from *Neufusicoccum batangarum*, the causal agent of the scabby canker of cactus pear [37]. It was toxic to tomato cuttings and young cork oak and holm oak leaves [36], and cactus pear cladode and tomato leaves by puncture assay [37].

Four benzopyrones namely (−)-*R*-mellein (**7**), (−)-*R*-3-hydroxymellein (**8**), 3*R*,4*R*-4-hydroxymellein (**9**), and 3*R*,4*S*-4-hydroxymellein (**10**) were isolated from the cultures of *Neofusicoccum parvum*, the pathogen of Botryosphaeria dieback of grapevine (*Vitis vinifera*). These four metabolites were phytotoxic to the leaves of grapevine by causing leaf necrosis. Among them, (−)-*R*-3-hydroxymellein (**8**) was the most phytotoxic [38]. Both 3*R*,4*R*-4-hydroxymellein (**9**) and 3*R*,4*S*-4-hydroxymellein (**10**) were also isolated from *Neufusicoccum batangarum*, the causal agent of the scabby canker of cactus pear. They induced necrotic lesions around the inoculation points of the cladodes of cactus pear and the leaves of tomato [39].

SMA93 (**11**) and *O*-methylated SMA93 (**12**) were isolated from *Fusarium proliferatum* ZS07, the endophytic fungus residing in the gut of long-hrned grasshopper (*Tettigonia chinensis*). They showed inhibitory activity on the radicle growth of *Amaranthus retrofleus* seedlings [22].

**Figure 2:**
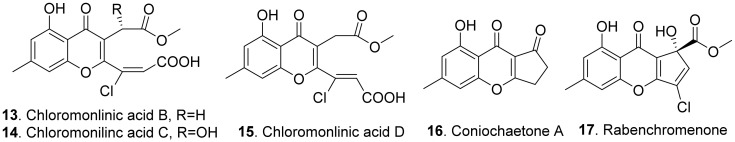
Structures of the phytotoxic benzo-γ-pyrones isolated from fungi.

The structures of the fungal phytotoxic benzo-γ-pyrones (chromenones) are shown in Figure 2. Chloromonilinic acids B (**13**), C (**14**), and D (**15**) were isolated from the liquid cultures of *Cochliobolus australiensis*, the leaf pathogen of the weed buffelgrass (*Pennisetum ciliare*). These three chloromonilinic acids were toxic to buffelgrass in a seedling elongation assay, with significantly delayed germination and dramatically reduced radicle growth [40].

Coniochaetone A (**16**) and rabenchromenone (**17**) were isolated from the culture filtrates of *Fimetariella rabenhorstii*, an oak-decline-associated fungus in Iran. They were toxic by causing a necrosis diameter in the range of 0.2–0.7 cm with a leaf puncture assay on tomato and oak leaves [41].

#### 2.1.2. Dibenzopyrones

Phytotoxic dibenzopyrones from fungi mainly include dibenzo-α-pyrones and dibenzo-γ-pyrones. Dibenzo-α-pyrones are a group of heptaketide coumarin derivatives that have a fused tricyclic nucleus.

**Figure 3:**
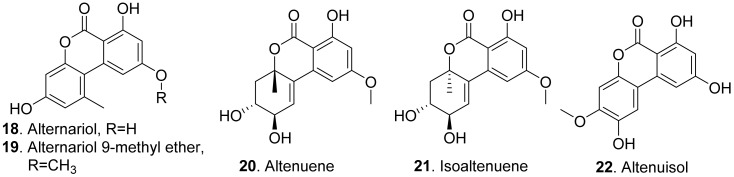
Structures of the phytotoxic dibenzo-α-pyrones isolated from fungi.

Many fungal dibenzo-α-pyrones possess a wide spectrum of biological activities such as cytotoxic, phytotoxic, and antimicrobial activities [42]. The structures of phytotoxic dibenzo-α-pyrones produced by the fungi from the genera *Alternaria* are shown in Figure 3.

Both alternariol (**18**) and alternuisol (**22**) were isolated from the cultures of *Alternaria* sp., the pathogen of the invasive weed *Xanthium italicum*. They inhibited shoot and root growth of *Pennisetum alopecuroides* and *Medicago sativa* by seedling growth assay [43]. Further studies on the mode of action showed that alternariol (**18**) and alternariol-9-methyl ether (also called AME, **19**) from *Alternaria alternata* inhibited the photosynthetic electron transport chain in isolated spinach chloroplasts [44].

Altenuene (**20**) and isoaltenuene (**21**), which were isolated from the marine-derived fungus *Alternaria* sp. P8, had inhibition on root and hypocotyl elongation of amaranth and lettuce at 200 μg/mL [27].

**Figure 4:**
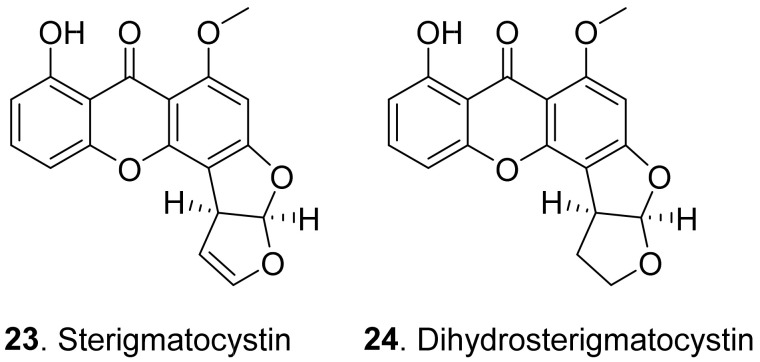
Structures of the phytotoxic dibenzo-γ-pyrones isolated from fungi.

Dibenzo-γ-pyrones (also called xanthones) are 9*H*-xanthen-9-one derivatives comprising a family of *O*-heterocycle symmetrical compounds with a dibenzo-γ-pyrone scaffold. Fungal dibenzo-γ-pyrones typically occur as completely aromatized dihydro-, tetrahydro-, or hexahydroderivatives. Two dibenzo-γ-pyrones (Figure 4) namely sterigmatocystin (**23**) and dihydrosterigmatocystin (**24**) exhibited potent herbicidal activity resulting in leaf necrosis and wilting. Dihydrosterigmatocystin (**24**) was more phytotoxic toward *Amaranthus retroflexus* than sterigmatocystin (**23**). Additionally, dihydrosterigmatocystin (**24**) also displayed remarkably herbicidal activity against other amaranthaceous plants including *Alternanthera philoxeroides*, *Amranthus spinosus*, and *Celosia argentea*. Analysis of the structure–herbicidal activity relationship indicated that the bifuranic ring played an important role in dibenzo-γ-pyrone (xanthone) phytotoxicity and the presence of a double bond in the furan ring could decrease phytotoxicity [45].

#### 2.1.3. Benzophenones

**Figure 5:**
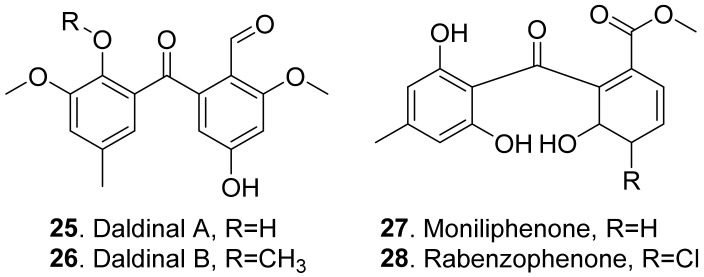
Structures of the phytotoxic benzophenones isolated from fungi.

Benzophenones share a common phenol-carbonyl-phenol skeleton. They are considered as the derivatives of xanthones [45]. The A-ring is derived from the shikimic acid pathway, and the B-ring is derived from the acetate-malonate pathway [46]. The structures of phytotoxic benzophenones from fungi are shown in Figure 5.

Two benzophenones named daldinalds A (**25**) and B (**26**) were isolated from *Daldinia concentrica*. Both metabolites showed inhibition on the root growth with a rice seedling assay [47].

Moniliphenone (**27**) and rabenzophenone (also called chloromoniliphenone, **28**) were isolated from the culture filtrates of *Fimetariella rabenhorstii*, an oak-decline-associated fungus in Iran. They were active by causing a necrosis diameter in the range of 0.2–0.7 cm with leaf puncture assay on tomato and oak leaves [41]. These two benzophenones were also isolated from the solid culture of *Alternaria sonchi*, the leaf pathogen of sowthistles (*Sonchus* spp.). Both metabolites were toxic to the leaves of couch-grass (*Elytrigia repens*) and sowthistle (*Sonchus arvensis*) by a punctured leaf disc assay [48].

#### 2.1.4. Naphthopyrones

**Figure 6:**
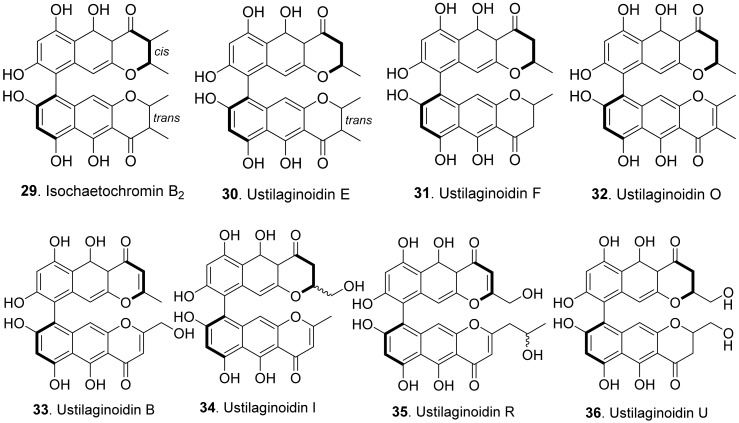
Structures of the phytotoxic bis-naphtho-γ-pyrones isolated from fungi.

Phytotoxic naphthopyrones from fungi belong to bis-naphthopyrones. Their structures are shown in Figure 6. Bis-naphtho-γ-pyrones were dimeric naphtha-γ-pyrones which are an important group of fungal polyketides with a variety of biological activities such as cytotoxic, antitumor, antimicrobial and phytotoxic activities [49]. Four bis-naphtho-γ-pyrones, namely isochaetochromin B_2_ (**29**), and ustilaginoidins E (**30**), F (**31**), and O (**32**), were isolated from rice solid cultures of *Ustilaginoidea virens* (teleomorph: *Villosiclava virens*), the pathogen of rice false smut disease. They displayed moderate inhibitory activity on the radicle elongation of rice seedlings. Among them, ustilaginoidin F (**31**) showed the strongest activity against the rice seeds [50]. Four other bis-naphtho-γ-pyrones with more polarity, namely ustilaginoidins B (**33**), I (**34**), R (**35**), and U (**36**), were later isolated from rice false smut balls. They exhibited inhibition on the radicle and germ growth of rice seedlings [51].

#### 2.1.5. Azaphilones

**Figure 7:**
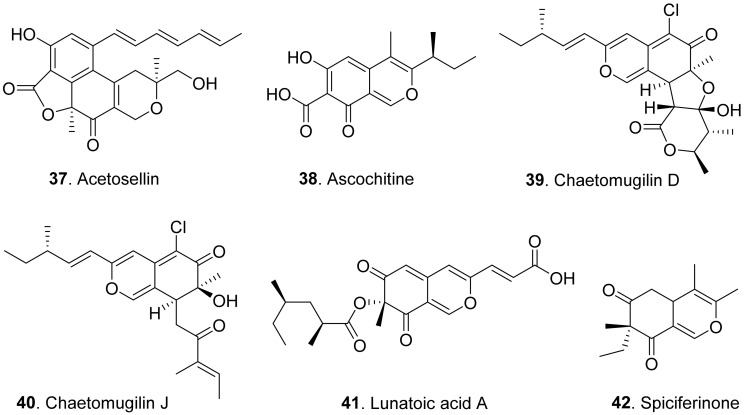
Structures of the phytotoxic azaphilones isolated from fungi.

Azaphilones (or called azaphilonoids) are a structurally variable family of fungal polyketide metabolites possessing a highly oxygenated pyranoquinone bicyclic core, usually known as isochromene, and a quaternary carbon center. They belong to a large group of fungal pigments, which turn red in the presence of primary amines due to an exchange of the pyran oxygen for nitrogen, arising from their affinity of the 4*H*-pyran nucleus to undergo substitution with primary amines to form the corresponding vinylogous γ-pyridones. Some fungal azaphilones showed phytotoxic activities. However, most of azaphilones have not been screened for their phytotoxic activities [52]. The structures of phytotoxic azaphilones from fungi are shown in Figure 7.

Acetosellin (**37**) was isolated from the mycelia of *Cercosporella acetosella*, the pathogen of leaf spots of the cosmopolitan weed (*Rumex acetosella*). It inhibited root elongation of *Lepidium sativum* and *Zea mais* at 6.4 × 10^−4^ M [53].

Ascochitine (**38**) was produced as a main phytotoxin from *Ascochyta fabae* and *A. pisi*, two pathogens that caused the so called ‘brown spots’ disease in broad bean and necrotic lesions on pea leaflets [54]. This compound was later isolated from the cultures of *Phoma clematidina*, the pathogen of leaf spot-wilt disease of *Clematis* sp. This metabolite was toxic to the leaves of *Clematis* sp. by leaf disc assay [55].

Chaetomugilins D (**39**) and J (**40**) were isolated from *Chaetomium globosum*, the endophytic fungus from the leaves of *Amaranthus viridis*. Both compounds showed phytotoxic activity in the lettuce (*Lactuca sativa*) seed germination assay [20].

Lunatoic acid A (**41**) was isolated from *Cladosporium oxysporum* DH14, the fungus residing in the gut of locust (*Oxya chinensis*). This metabolite exhibited significant inhibition against radicle growth of *Amaranthus retroflexus* seedlings [24].

Spiciferinone (**42**) was isolated from the culture filtrates of *Cochliobolus spicifer*, the pathogen of leaf spot disease in Gramineae. This metabolite was phytotoxic to wheat cotyledons by using protoplast viability assay [56].

#### 2.1.6. Naphthalene Derivatives

**Figure 8:**
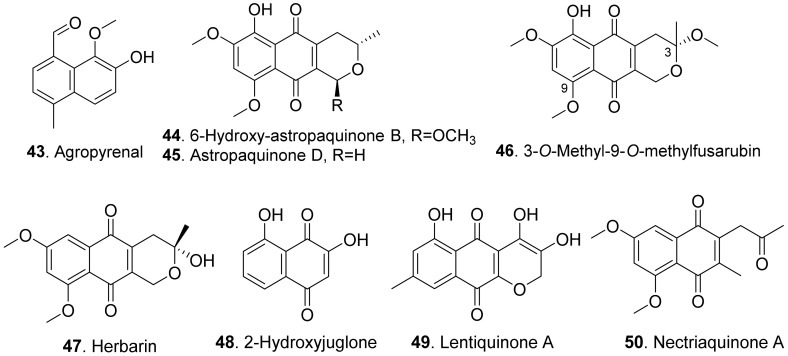
Structures of the phytotoxic naphthol and naphthoquinone congeners isolated from fungi.

Phytotoxic fungal naphthalene derivatives include naphthols, naphthoquinones, and naphthalenones. One naphthol and seven naphthoquinones with phytotoxicity were found in fungi. Their structures are shown in Figure 8.

Agropyrenal (**43**), a naphthol, was isolated from the liquid cultures of *Ascochyta agropyrina* var. *nana*. When the leaves of several weed plants (i.e., *Mercurialis annua*, *Chenopodium album* and *Setaria viridis*) were assayed, agropyrenal (**43**) was proved to be phytotoxic by causing the appearance of necrotic lesions [57].

Three naphthoquinone derivatives, 6-hydroxy-astropaquinone B (**44**), astropaquinone D (**45**) and 3-*O*-methyl-9-*O*-methylfusarubin (**46**) were isolated from *Fusarium napiforme*, an endophytic fungus isolated from the mangrove plant, *Rhizophora mucronata*. They inhibited growth of lettuce seedlings at concentration of 30 μg/mL [58].

Herbarin (**47**) and nectriaquinone A (**50**) isolated from *Nectria pseudothichia* 120-1 NP inhibited the growth of lettuce (*Lactuca sativa* L.) seedlings. Herbarin (**47**) showed stronger phytotoxicity than nectriaquinone A (**50**) [59].

Further, 2-hydroxyjuglone (**48**) was isolated from the culture broth of *Ceratocystis fimbriata* f.sp. *platani*, the canker pathogen of plane tree (*Platanus orientalis*). This compound induced large necrotic lesions in stem explants of plane tree as was observed in vivo [60].

Lentiquinone A (**49**) was isolated from *Ascochyta lentis*, the pathogen of lentil (*Lens culinaris*). It exhibited a strong phytotoxicity to the punctured leaves and seed germination of host and non-host plants [61].

**Figure 9:**
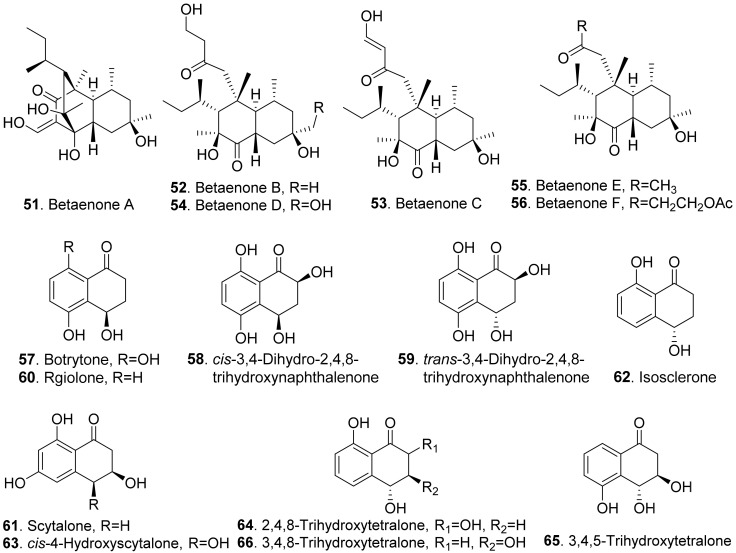
Structures of the phytotoxic naphthalenone congeners isolated from fungi.

The structures of phytotoxic naphthalenone derivatives from fungi are shown in Figure 9. Six polysubstituted octahydro-naphthalen-1-ones, named betaenones A–F (**51**-**56**) were isolated from *Phoma betae*, the causal agent of leaf spot disease of sugar beet. Among these metabolites, betaenone C (**53**) exhibited the highest phytotoxic activity to cause wilting of sugar beet at 50 μg/mL, and highest root growth inhibitory effect (89%) against rice seedlings at 37 μg/mL [62,63].

Four naphthalenones were isolated from *Botrytis fabae*, the pathogen of faba bean (*Vicia faba*) by displaying clear chocolate spot symptom. They were named as botrytone (**57**), *cis*- and *trans*-3,4-dihydro-2,4,8-trihydroxynaphthalene-1(2*H*)-ones (**58** and **59**), and regiolone (**60**) [64].

Scytalone (**61**) and isosclerone (**62**) were two phytotoxic metabolites produced by both *Phaeoacremonium aleophilum* (syn. *P. miimum*) and *P. chlamydospora*, the causal agent of esca disease [65]. Scytalone (**61**) was also isolated from *Raffaelea quercivora* which was the pathogen of Japanese oak wilt disease [66], and from *Ceratocystis imbriata* f.sp. *platani* which was the canker pathogen of plane tree (*Platanus occidentalis*) [60]. This compound exhibited weak phytotoxic activity by inhibiting root growth of lettuce seedlings [66], and caused significant leaf necrosis on the cutting seedlings of plane tree [60]. Phytotoxic scytalone (**61**), isolerone (**62**), and *cis*-4-hydroxyscytalone (**63**) were also isolated from *P. aleophilum* by another research group [67].

Both 2,4,8-trihydroxytetralone (**64**) and 3,4,8-trihydroxytetralone (**66**) from *Phaeoacremonium aleophilum* inhibited callus growth of grapevine [68]. 3,4,5-Trihydroxytetralone (**65**) and 3,4,8-trihydroxytetralone (**66**), which were isolated from the fungus *Hypoxylon mammatum*, the stem canker pathogen of aspen (*Populus* sp.), were toxic to the aspen leaves by leaf assay [69].

**Figure 10:**
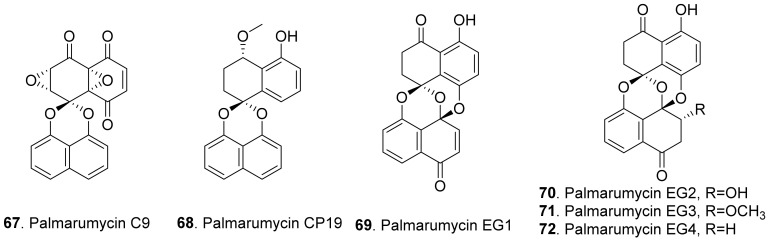
Structures of the phytotoxic spirobisnaphthalenes isolated from fungi.

Spirobisnaphthalenes (also called bisnaphthospiroketals or spirodioxynaphthalenes) have a unique structure in which two napthalenes are connected by spiroketal or binaphalene spiroether. Most of these metabolites isolated from fungi exhibit significant phytotoxic, antimicrobial, and cytotoxic activities to show great potential applications in medicine and agriculture [70,71]. The structures of phytotoxic spirobisnaphthalenes from fungi are shown in Figure 10.

Palmarumycin C9 (**67**) was isolated from the cultures of *Coniothyrium* sp. to show herbicidal activity by inhibiting the growth of the cultured cells of algae, lemna, and corn [72].

Palmarumycins CP19 (**68**), EG1 (**69**), EG2 (**70**), EG3 (**71**) and EG4 (**72**) were isolated from the mycelial fermentation cultures of the endophytic fungus *Edenia gomezpompae* from the plant *Callicarpa acuminata* (Verbenaceae). Among them, palmarumycins EG1-EG4 (**69**–**72**) inhibited seed germination of *Amaranthus hypochondriacus*, *Solanum lycopersicum*, and *Echinochloa crus-galli* by more than 50%. Palmarumycins CP19 (**68**), EG2 (**70**), and EG3 (**71**) inhibited root elongation of *A. hypochondriacus* and *E. crus-galli* seedlings by more than 50%. These five palmarumycins also inhibited the oxygen consumption of three plant seedlings [73].

#### 2.1.7. Anthraquinones

**Figure 11:**
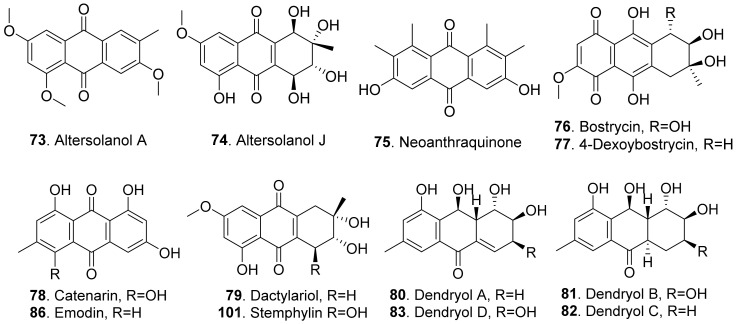

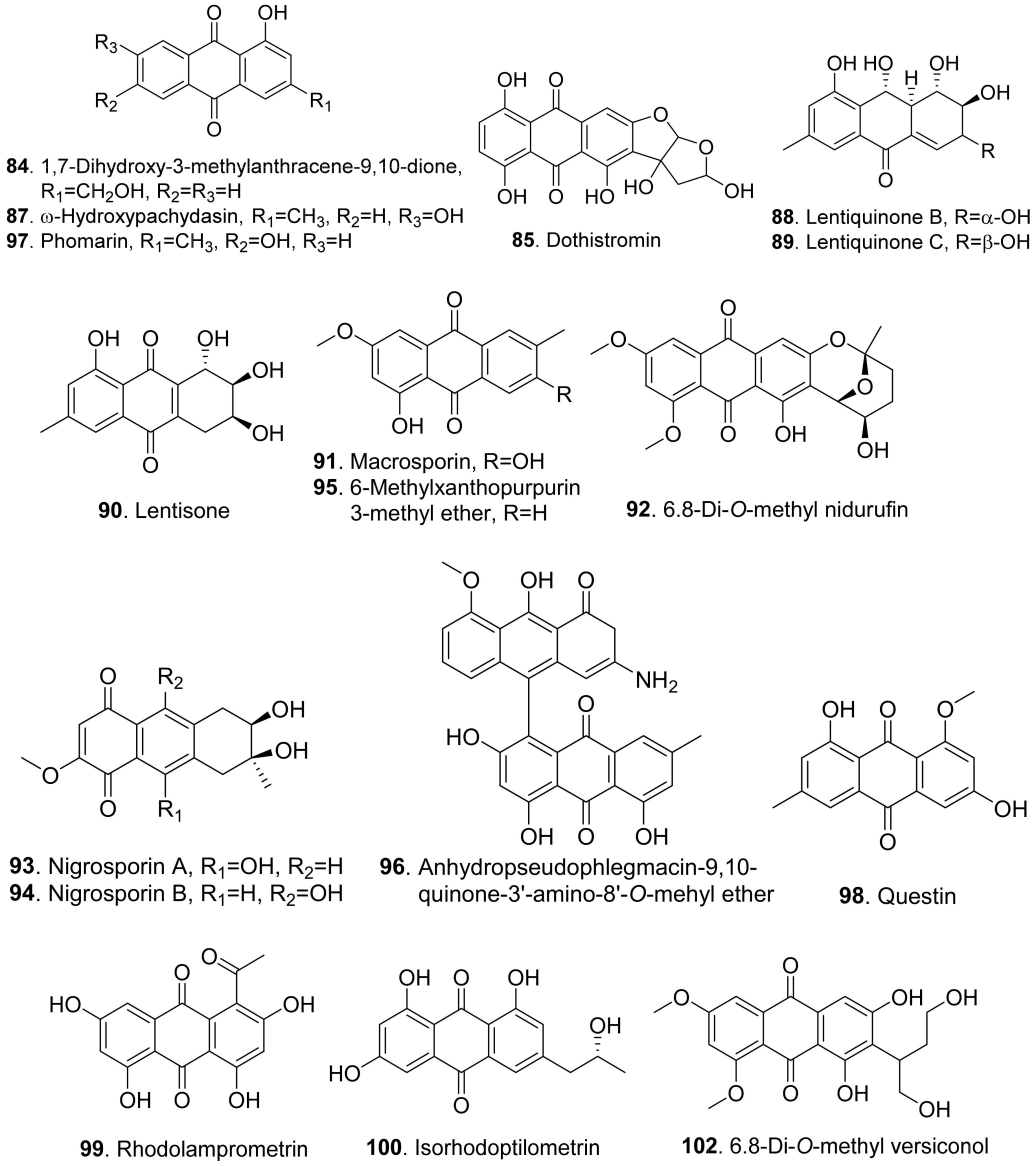
Structures of the phytotoxic anthraquinones isolated from fungi.

Anthraquinones are a group of polyketides containing eight C2 units, which generates in turn with three aldol type condensations of the carbon skeleton of anthraquinones except for the two carbonyl oxygens of the central ring [74]. The structures of fungal phytotoxic anthraquinones are shown in Figure 11.

Two anthraquinones, namely altersolanols A (**73**) and J (**74**), were isolated from the pathogen *Phomopsis foeniculi* (teleomorph: *Diaporthe angelicae*) of fennel (*Foeniculum vulgare*). They showed a modulated phytotoxicity on the detached tomato leaves [75]. Altersolanol A (**73**) was also isolated from *Alternaria porri*. This compound inhibited growth of lettuce and stone-leek seedlings [76].

Neoanthraquinone (**75**) was isolated from *Neofusicoccum luteum*, the causal agent of Botryosphaeria dieback in Australia. Neoanthraquinone (**75**) showed the obvious toxic effect by causing severe shriveling and withering on grapevine by leaf assay [37].

Both bostrycin (**76**) and 4-deoxybostrycin (**77**) were isolated from the culture filtrates of *Alternaria eichhorniae*, the pathogen of the water hyacinth (*Eichhornia crassipes*). These two compounds induced leaf necrosis on water hyacinth [77]. Bostrycin (**76**) was also isolated from *Alternaria alternata*, another pathogen of water hyacinth [78].

Catenarin (**78**) was produced by the necrotrophic fungus *Pyrenophora tritici-repentis* (anamorph: *Drechslera tritici-repentis*), the causal agent of tan spot foliar pathogen of wheat. Catenarin (**78**) induced necrosis on the leaves of wheat. It also infected wheat kernels by causing a red discoloration known as red smudge [79].

Dactylariol (**79**), macrosporin (**91**), and stemphylin (**101**) were produced by *Stemphylium botryosum*, the pathogen inducing a destructive disease of lettuce. They caused sunken necrotic brown lesions of lettuce leaves by leaf puncture assay [80].

Dendryols A–D (**80**–**83**) were produced by *Dendryphiella* sp., the fungus isolated from an infected sample of the paddy weed *Eleocharis kuroguwai* (Cyperaceae) in Japan. When tested for the phytotoxic activity by a leaf-puncture assay on weeds (kuroguwai, barnyardgrass, and velvetleaf) and cultivated crops (rice, corn, and cowpea), dendryols A–D (**80**–**83**) showed toxicity only against barnyardgrass and the nercrotic area appeared to be dose-dependent, and dendryol A (**83**) caused similar necrosis only on velvetleaf [81].

Moreover, 1,7-dihydroxy-3-methylanthracene-9,10-dione (**84**), ω-hydroxypachybasin (**87**), lentisone (**90**), and phomarin (**97**) were isolated from the culture filtrates of *Ascochyta lentis*, the pathogen of lentil (*Lens culinaris*). These compounds caused severe necrosis to the punctured leaves and inhibited seed germination of lentil [61].

Dothistromin (**85**) was isolated as the main phytotoxin produced by *Dothistroma pini*, the pathogen by causing necrotic disease characterized by the formation of red bands on the infected needles of *Pinus radiata* and other pines [82].

Emodin (**86**) has been identified in many fungi [83] and plants [84,85]. Emodin (**86**) isolated from the coprophilous fungus *Guanomyces polythrix* showed inhibition on seedling radicle growth of weeds *Amaranthus hypochondriacus* and *Echinochloa crusgalli* [86].

Lentiquinones B (**88**) and C (**89**) were isolated from *Ascochyta lentis*, the pathogen of lentil (*Lens culinaris*). Both compounds caused severe leaf necrosis when applied to the punctured leaves of host and non-host plants. [61].

Lentisone (**90**) was an anthraquinone produced by *Ascochyta lentis*. This metabolite was phytotoxic to lentil (*Lens culinaris*), and was light-dependent [87].

Macrosporin (**91**) and 6-methylxanthopurpurin 3-methyl ether (**95**) were isolated from *Alternaria bataticola* which was the causal fungus to black spot of sweet potato [88]. Macrosporin (**91**) was also isolated from *Stemphyfium botryosum*. It was toxic to lettuce by leaf punctrue assay [80].

Both 6,8-di-*O*-methyl nidurufin (**92**) and 6,8-di-*O*-methyl versiconol (**102**) were isolated from the solid cultures of *Penicillium purpurogenum* derived from soil. These two compounds inhibited root and hypocotyl growth of radish seedlings at 100 μM [26].

Both nigrosporins A (**93**) and B (**94**) were isolated from the culture filtrates of *Nigrospora oryzae*. The two compounds showed significant necrotic effects in leaf puncture assay on green foxtail (*Setaria viridis*), barnyardgrass (*Echinochloa crus-galli*), velvetleaf (*Abutilon theophrasti*), corn (*Zea mays*), and cowpea (*Vigna sesquipedalis*). They also had an inhibition on root elongation of lettuce seedlings [89].

An anhydropseudophlegmacin-quinone named anhydropseudophlegmacin-9,10-quinone-3′-amino-8′-*O*-methyl ether (**96**) was isolated from the filtrate of *Phoma herbarum* FGCC#54. This compound was a dimeric anthraquinone. It was toxic to the weeds *Parthenium hysterophorus*, *Lantana camara* and *Hyptis suaveolens* to show its herbicidal potential against the prominent weeds [90].

Questin (**98**) and isorhodoptilometrin (**100**) were isolated from the endophytic fungus *Aspergillus* sp. YL-6 residing in *Pleioblastus amarus*. Both anthraquinones inhibited the seed germination and seedling growth of wheat (*Triticum aestivum*) and radish (*Rahanus sativus*). Questin (**98**) obviously inhibited shoot and root elongation of wheat seedlings [91].

Rhodolamprometrin (**99**) was isolated from *Fusarium proliferatum* ZS07, the endophytic fungus residing in the gut of long-horned grasshopper (*Tettigonia chinensis*). This compound exhibited inhibitory activity on the radicle growth of *Amaranthus retrofleus* seeds to show its potential as a biocontrol agent in agriculture [22].

#### 2.1.8. Perylenequinonoids

**Figure 12:**
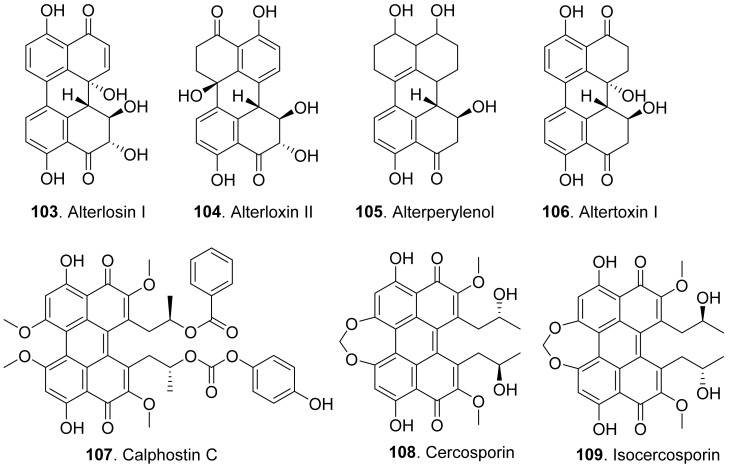

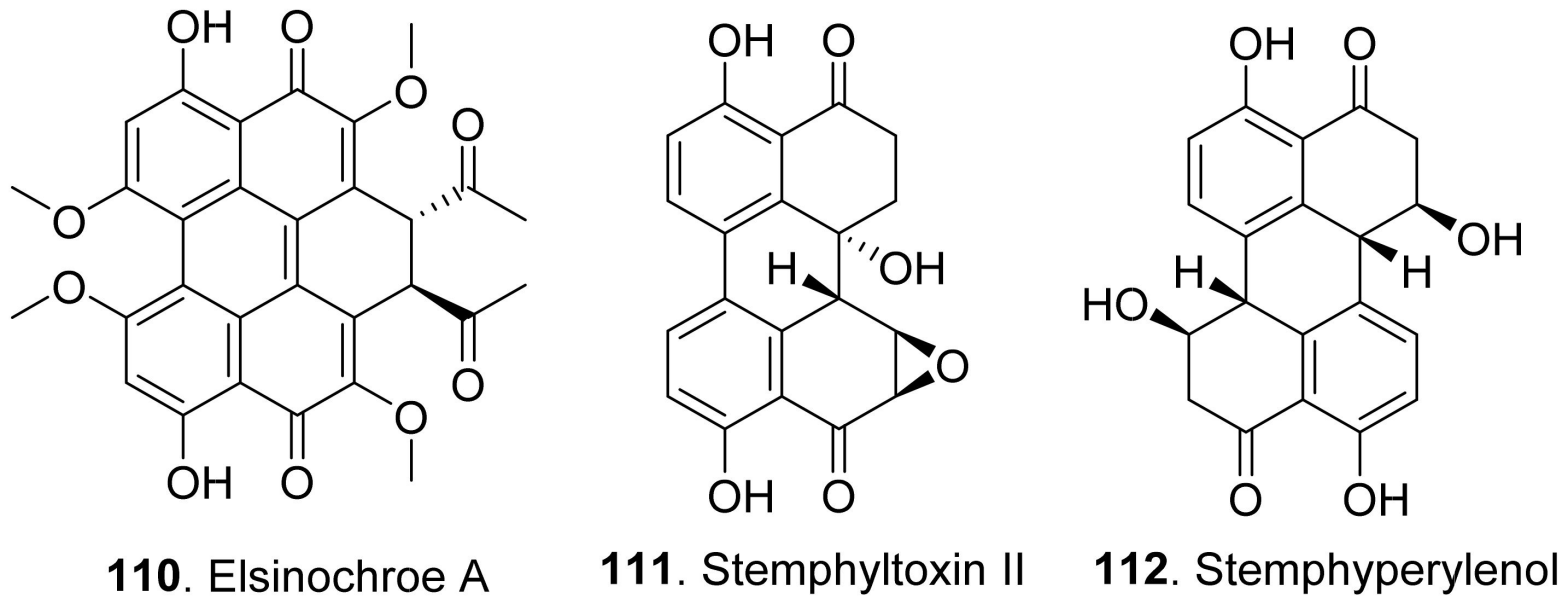
Structures of the phytotoxic perylenequinonoids isolated from fungi.

Perylenequinonoids are a class of aromatic polyketides characterized by a pentacyclic conjugated chromophore. Fungal perylenequinones are the photoactivated phytotoxins which act by absorbing light energy and generating reactive oxygen species that damage host plant cells [92]. The structures of phytotoxic perylenequinonoids from fungi are shown in Figure 12.

Alterlosins I (**103**) and II (**104**) were isolated from the cultures of *Alternaria alternata*, the pathogen of spotted knapweed (*Centaurea maculosa*), a major weed pest in rangelands of the northwestern United States and southwestern Canada. Both metabolites induced necrotic lesions on knapweed by a leaf puncture assay. Alterlosin I (**103**) induced larger necrotic lesions compared to the small flecks induced by alterlosin II (**104**) [93].

Four perylenequinone congeners alterperylenol (also called alteichin, **105**), altertoxin I (**106**), stemphyltoxin II (**111**), and stemphyperylenol (**112**) were isolated from *Alternaria cassiae*, the pathogen of sicklepod (*Cassia obtusifolia*). They were toxic to sicklepod, corn, crabgrass, timothy and soybean to cause leaf necrosis by leaf puncture assay. Stemphyperylenol (**112**) was a selective toxin for crabgrass, while altertoxin I (**106**) was selective for corn B73 [94].

Three perylenequinonoids named alterperylenol (**105**), altertoxin I (**106**) and stemphyperylenol (**112**) were isolated from the marine-derived fungus *Alternaria* sp. P8. They showed inhibition on root and hypocotyl elongation of amaranth and lettuce seedlings at 50 μg/mL [27]. 

Calphostin C (**107**) was isolated from plant pathogen *Cladosporium cladosporioides*. This metabolite was a protein kinase C (PKC) inhibitor by competing at the binding site for diacyglycerol and phorbol esters. Specific inhibitor of PKC would be very useful for calphostin C (**107**) as the pharmacological tool and potential drug [95].

Cercosporin (**108**) was isolated from cultures of *Cercospora nicotianae*, and was tested for toxic effects on suspension-cultured cells of tobacco. Cercosporin (**108**) was toxic to tobacco cells only when it was incubated under the light [96]. It was found that cercosporin (**108**) can be produced by a few pathogenic fungi in the genus *Cercospora*. It was toxic to plants by the generation of activated oxygen species, particularly singlet oxygen. *Cercospora* fungi penetrate host tissues through the stomata and colonize the intercellular spaces. Production of the membrane-damaging cercosporin (**108**) would allow for cell breakdown and leakage of nutrients required by the fungi for growth and sporulation in the host plant [97]. Isocercosporin (**109**) was isolated from *Scolecotrichum graminis*, the causal fungus of a leaf streak disease of orchardgrass. This metabolite was higher toxic than cercosporin (**108**) by lettuce seedling growth assay [98].

Elsinochrome A (**110**) was isolated from *Stagonospora convolvuli*, the biocontrol fungus to bindweed (*Convolvulus arvensis*). This metabolite showed inhibition on the root elongation of tomato by seedling growth assay, and toxic to bindweed and grapevine leaves by leaf-wounded assay [99].

#### 2.1.9. Aromatic Macrolides

**Figure 13:**
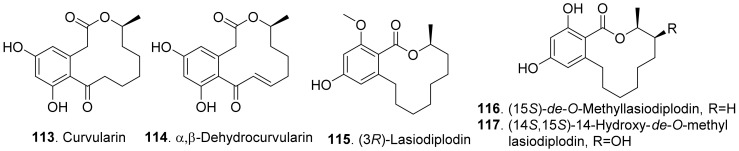

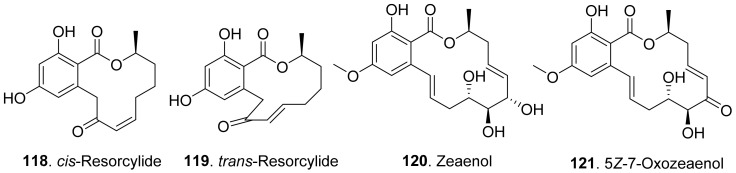
Structures of the phytotoxic aromatic macrolides isolated from fungi.

Aromatic macrolides are a class of fungal polyketides possessing a macrolide core structures fused into an aromatic ring. The typical metabolites are benzenediol lactones. They have various biological activities such as phytotoxic, cytotoxic, and nematicidal activities. The structures of phytotoxic aromatic macrolides from fungi are shown in Figure 13.

Curvularin (**113**) and α,β-dehydrocurvularin (**114**) were isolated from the cultures of *Curvularia intermedia*, the leaf pathogen of *Pandanus amaryllifolius*. Both metabolites were toxic to lettuce (*Lactuca sativa*) and bentgrass (*Agrostis stolonifera*) with seed germinatin assay [100]. In addition, α,β-dehydrocurvularin (**114**) was isolated from the culture filtrates of *Alternaria zinnia*, the fungus causing leaf necrosis of *Xanthium occidentale*. It was toxic to the test plants by using leaf puncture assay [101]. α,β-Dehydrocurvularin (**114**) was also isolated from *Nectria galligena*, the apple canker pathogen in Chile. This compound significantly reduced elongation and epicotyl growth of lettuce seedlings [102].

(3*R*)-Lasiodiplodin (**115**), which was isolated from *Botryosphaeria rhodina*, inhibited photophosphorylation and electron transport chain in thylakoids. (3*R*)-Lasiodiplodin (**115**) behaved as a Hill reaction inhibitor of the oxygen-evolving complex on chloroplasts. It also interacted at coupling factor 1 (CF_1_) by inhibiting CF_1_ Mg^2+^-ATPase activity [103].

Two β-resorcylic acid derivatives namely (15*S*)-*de*-*O*-methyllasiodiplodin (**116**) and (14*S*,15*S*)-14-hydroxy-*de*-*O*-methyllasiodiplodin (**117**) were isolated from rice fermentation cultures of the endophytic fungus *Lasiodiplodia theobromae* derived from the mangrove plant *Xylocarpus granatum*. Both compounds inhibited root elongation of *Digitaria ciliaris* [104].

Both *cis*-resorcylide (**118**) and *trans*-resorcylide (**119**) isolated from *Penicillium* sp. inhibited root elongation of the seedlings of Chinese cabbage, lettuce, and rice, whereas *trans*-Resorcylide (**119**) was more phytotoxic than *cis*-resorcylide (**118**) [105].

Zeaenol (**120**) and 5*Z*-7-oxozeaenol (**121**) were isolated from the fermentation broth of *Cladosporium oxysporum* DH14, a locust-associated fungus. Both compounds exhibited significantly phytotoxic activities against the radicle growth of *Amaranthus retroflexus* seedlings with IC_50_ values of 8.16 μg/mL and 4.80 μg/mL, respectively [24].

### 2.2. Aliphatic Polyketides

Aliphatic polyketides usually have either linear or macrocyclic non-aromatic carbon frameworks, many of which are lactones. Aliphatic polyketides mainly include furan and furanone analogues, aromatic-free pyrones, furopyrans, macrolides, sorbicillinoids, and linear polyketides.

#### 2.2.1. Simple Furan and Furanone Analogues

**Figure 14:**
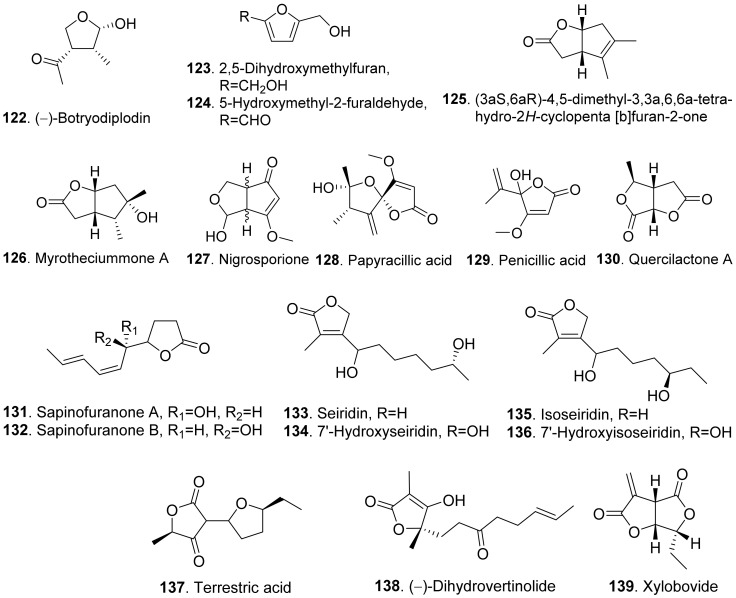
Structures of the phytotoxic furan and furanone analogures isolated from fungi.

The structures of phytotoxic furan and furanone analogues from fungi are shown in Figure 14. (−)-Botryodiplodin (**122**) was isolated from the cultures of *Botryodiplodia thebromae*, the pathogen of soybean charcoal rot disease. (−)-Botryodiplodin (**122**) was a simple lactol analogue which was toxic to soybean and duckweed (*Lemna pausicostata*) [106]. This compound has been synthesized by using stereoselective radical cyclizations of acyclic esters and acetals [107].

Two furan derivatives, namely 2,5-dihydroxymethylfuran (**123**) and 5-hydroxymethylfuraldehyde (**124**), were isolated from the cultures of *Stilbocrea macrostoma*, the fungal pathogen of the tree *Quercus brantii,* by inducing wood necrosis [108].

(3aS,6a*R*)-4,5-Dimethyl-3,3a,6,6a-tetrhydro-2*H*-cyclopenta [b]furan-2-one (**125**) and myrotheciumone A (**126**) were isolated from fermentation broth of endophytic fungus *Xylaria curta* 92092022. Both compounds inhibited hypocotyl and root growth of lettuce seedlings [109].

Nigrosporione (**127**) was isolated from *Neofusicoccum luteum*, the causal agent of Botryosphaeria dieback in Australia. It showed the phytotoxic effect by causing severe shriveling and withering on grapevine by leaf assay [37].

Papyracilic acid (**128**) was a 1,6-dioxaspiro[4,4]nonene isolated from the solid culture of *Ascohyta agropyrina* var. *nana*, the leaf pathogen of quack grass (*Elytrigia repens*). This compound was toxic to host plant and a number of non-host plants of the fungus. It was considered as the potential mycoherbicide for control of *E. repens* [110].

Penicillic acid (**129**) from *Malbranchea aurantiaca* showed significant inhibition of radicle growth of *Amaranthus hypochondriacus* seedlings with IC_50_ value of 3.86 μM [111].

Quercilactone A (**130**) was isolated from *Raffaelea quercivora*, the pathogen of Japanese oak wilt disease. This compound exhibited weak phytotoxic activity by inhibiting root growth of lettuce seedlings [66].

Sapinofuranones A (**131**) and B (**132**), belonging to 5-substituted dihydrofuranones, were isolated from liquid cultures of *Sphaeropsis sapinea*, the pathogen to cause a wide range of disease symptoms on conifers such as *Cupressus macrocarpa* and *C. sempervirens*. Both metabolites were diastereomers of each other. Bioassay of sapinofuranones A (**131**) and B (**132**) gave epinasty and brown discoloration on petioles of tomato leaves, sapwood stain on inner cortical tissues of the stem of cypress seedlings, and yellowing and needle blight on pine seedlings [112].

Seiridin (**133**) and isoseiridin (**135**), belonging to Δα,β-butenolides, were produced by the cultures of *Seiridium cardinal*, *S. cupressi*, and *S. unicorne*. Seiridin (**133**) showed inhibition on lettuce germination, and isoseiridin (**135**) did not [113]. 7′-Hydroxyseiridin (**134**) and 7′-hydroxyisoseiridin (**136**) were also isolated from the cultures of *Seiridium cardinal*, *S. cupressi*, and *S. unicorne*. Both hydroxylation products of seiridin (**133**) and isoseiridin (**135**) produced necrotic and chlorotic symptoms on the cuttings of host plants (*Cupressus sempervirens* var. *pyramidalis*, *C. arizonica*, and *C. macrocarpa*) and non-host plants (*Lycopersicon esculentum* and *Phaeolus valgaris* var. *arueus*) [114].

Terrestric acid (**137**) was isolated from the culture broth of *Magnaporthe oryzae* (anamorph: *Pyricularia oryza*). It inhibited root and leaf growth of rice seedlings at 300 μg/mL [115].

(−)-Dihydrovertinolide (**138**) belonged to α-furanone isolated from *Clonostachys rosea* B5-2. This compound displayed phytotoxic activity against lettuce seedlings at concentration of 50 μg/mL [116].

Xylobovide (**139**) was isolated from the culture broth of *Xylaria obovata*. This metabolite inhibited seed germination of *Eragrostis tef* at 50–100 μg/mL [117].

#### 2.2.2. Aromatic-Free Pyrones

**Figure 15:**
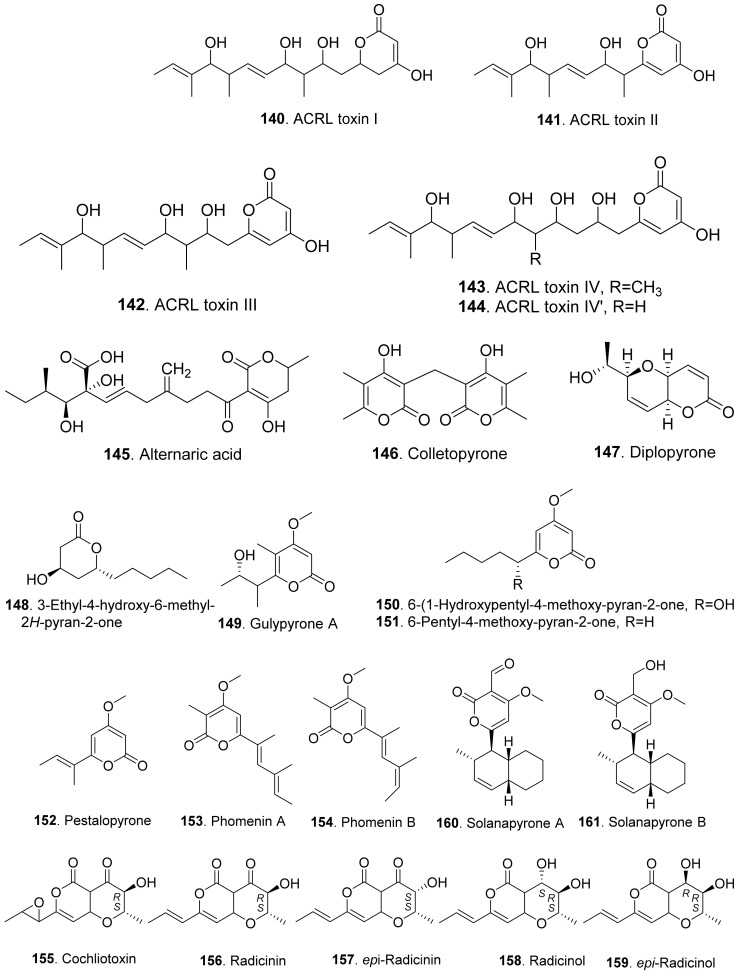
Structures of the phytotoxic aromatic-free α-pyrones isolated from fungi.

Phytotoxic aromatic-free pyrones include α-pyrones and γ-pyrones. Most of them belong to α-pyrones. The structures of phytotoxic aromatic-free α-pyrones from fungi are shown in Figure 15. ACRL toxins I (**140**), II (**141**), III (**142**), IV (**143**), and IV’ (**144**) were isolated from the culture broth of *Alternaria citri*, the fungal pathogen causing brown spot disease of rough lemon (*Citrus jambhiri*) and Rangpur Lime (*Citrus limonia*). They were toxic to the host plants rough lemon and Rangpur Lime by leaf puncture assay and electrolyte leakage assay. These ACRL toxins were considered as the host-specific phytotoxins [118,119].

Alternaric acid (**145**) was isolated from the culture filtrates of *Alternaria solani*, the pathogen of early blight and collar rot diseases on tomato plants. Alternaric acid (**145**) was toxic to tomato seedlings [120].

Colletopyrone (**146**) was isolated from *Colletotrichum nicotianae*. This compound caused brown necrotic spots on young tobacco leaves [121].

Simple α-pyrones are often lactone derivatives of fatty acids. Diplopyrone (**147**) was a phytotoxic metabolite of *Diplodia corticola* [122] and *Diplodia mutila* [123], which were phytopathopagenic fungi causing different forms of cork oak canker on *Quercus suber* with heavy economic losses. Diplopyrone (**147**) was toxic to the cuttings of cork oak and tomato by causing necrosis and wilting. The absolute configuration of diplopyrone (**147**) was determined by vis-à-vis comparison of experimental and simulated spectra [124].

Further, 3-ethyl-4-hydroxy-6-methyl-2*H*-pyran-2-one (**148**) isolated from *Raffaelea quercivora*, the pathogen of Japanese oak wilt disease, had weak phytotoxic activity by inhibiting root growth of lettuce seedlings [66].

Gulypyrone A (**149**) from *Diapothe gulyae*, the pathogen of sunflower (*Helianthus annuus*), caused leaf necrosis on sunflower plantlets [125].

Then, 6-(1-hydroxypentyl)-4-methoxypyran-2-one (**150**), 6-pentyl-4-methoxy-pyran-2-one (**151**) and pestalopyrone (**152**) were isolated for *Pestalotiopsis guepinii*. They all showed phytotoxic activity on some non-host plants (i.e., *Convolvulus arvensis*, *Mercurialis annua*, *Chenopodium album* and *Ailanthus altissima*) by leaf puncture assay [126].

Pestalopyrone (**152**) was a pentaketide phytotoxin isolated from *Pestalotiopsis guipinii*, the pathogen to cause twig of hazelnut (*Corylus avellana*). This compound was toxic to a few non-host plants such as *Cirsium arvense*, *Sonchus oleraceus*, and *Chenopodium album* by causing extensive necrosis on the test plant leaves [127].

Phomenins A (**153**) and B (**154**) were polypropionate α-pyrones produced by *Phoma tracheiphila*, the pathogen of the destructive disease of lemon groves in the Mediterranean area. Only phomenin A (**153**) showed phytotoxicity on tomato cutting while both phomenins A (**153**) and B (**154**) exhibited zootoxcity against brine shirimp (*Artemia salina* L.) larvae [128].

Radicinin (**156**), radicinol (**158**) and *epi*-radicinol (**159**) were isolated from cultures of *Alternaria radicina* grown on carrot slices. Both radicinin (**156**) and *epi*-radicinol (**159**) reduced root elongation of germinating carrot seeds [129]. Five phytotoxic radicinin derivatives named cochliotoxin (**155**), radicinin (**156**), *epi*-radicinin (**157**), radicinol (**158**), and *epi*-radicinol (**159**) were also isolated from the liquid culture of *Cochiliobolus australiensis*, the foliar pathogen of buffelgrass (*Pennisetum ciliaris*). The phytotoxic metabolites could potentially be used as natural herbicides in an integrated pest management strategy against the weed buffelgrass [130]. In addition, radicinin (**156**) was isolated from *Curvularia* sp. FH01 as the endophytic fungus in the gut of *Atractomorpha sinensis*. This compound exhibited significant phytotoxic activity against the radical growth of *Echinochloa crusgalli* [131]. Radicinin (**156**) was also isolated from *Fusarium proliferatum* ZS07, the fungus residing in the gut of long-horned grasshopper (*Tettigonia chinensis*). It showed inhibitory activity on the radicle growth of *Amaranthus retrofleus* seedlings at 100 μg/mL [22].

Solanapyrones A (**160**) and B (**161**) were isolated from the culture filtrates of *Alternaria solani*, the causal organism of early blight disease of tomato and potato. Both metabolites induced leaf necrotic lesion of the host plants [132]. Solanapyrone A (**160**) was later isolated from the culture filtrates of *Ascochyta rabiei* grown in the Czapek-Dox medium supplemented with seed aqueous extract of host plant chickpea. Solanapyrone A (**160**) was toxic to the cultured cells of chickpea [133].

**Figure 16:**
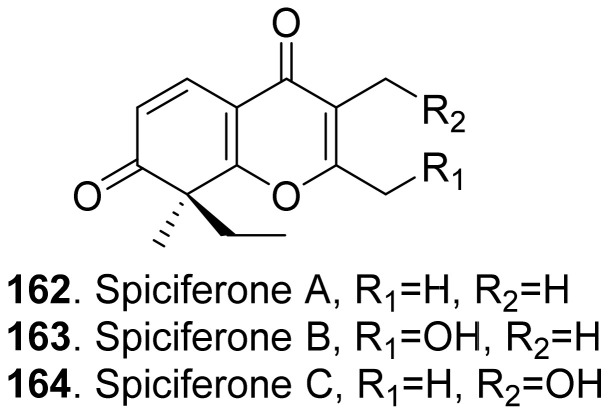
Structures of the phytotoxic aromatic-free γ-pyrones isolated from fungi.

Three phytotoxic aromatic-free γ-pyrones (Figure 16) namely spiciferones A (**162**), B (**163**) and C (**164**) were isolated from the fungus *Cochliobolus spicifer*. Among them, spiciferone A (**162**) was the most toxic to wheat cotyledon protoplasts, spiciferone C (**164**) was the least, and spiciferone B (**163**) had no activity. This indicated that the substitution on the γ-pyrone ring of spiciferone A (**162**) affected its phytotoxicity, and the methyl at C-2 was also essential to its phytotoxicity [134,135].

#### 2.2.3. Furopyran and Pyranopyran Analogues

**Figure 17:**
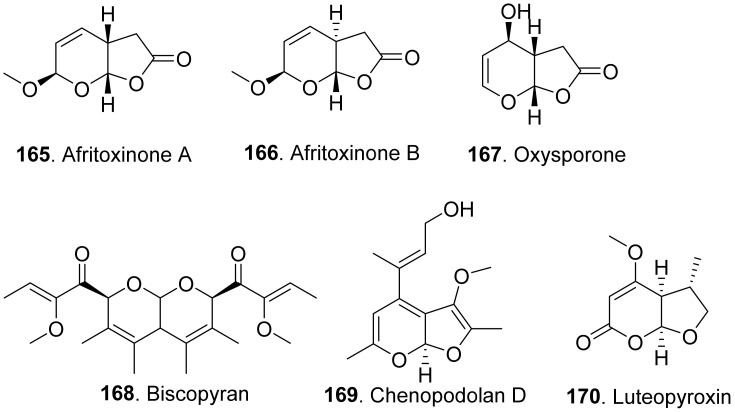
Structures of the phytotoxic furopyran and pyranpyran analogues isolated from fungi.

Phytotoxic furanpyran and pyranpyran analogues from fungi with their structures are shown in in Figure 17. Three dihydrofuropyran-2-ones afritoxinones A (**165**) and B (**166**), and oxysporone (**167**) were isolated from *Diplodia africana*, the causal agent of branch dieback on *Juniperus phoenicea*. Three compounds showed phytotoxic activity on host (*Phoenicean juniper*) and non-host plants (holm oak, cork oak and tomato) by cutting and leaf puncture assays. Among them, oxysporone (**167**) was the most phytotoxic compound [136].

Biscopyran (**168**) was a phytotoxic hexasubstituted pyranopyran isolated from the liquid culture filtrates of *Biscogniauxia mediterranea*, the pathogen of cork oak (*Quercus suber*). This compound caused epinasty on cork oak cuttings, and wilting on non-host tomato [137].

Chenopodolan D (**169**) was isolated from *Phoma chenopodiicola*, the pathogen of *Chenopodium album*. Chenopodolan D (**169**) was toxic to six weedy plants, namely *Stellaria media*, *Urtica dioica*, *Sonchus arvensis*, *Parietaria officinalis*, *Lactuca serriola*, and *Helianthus annuus* by leaf puncture assay [138].

Luteopyroxin (**170**) was isolated from *Neofusicoccum luteum*, the causal agent of Botryosphaeria dieback in Australia. This compound showed the phytotoxic effect by causing severe shriveling and withering on grapevine by leaf assay [37].

#### 2.2.4. Macrolide Analogues

**Figure 18:**
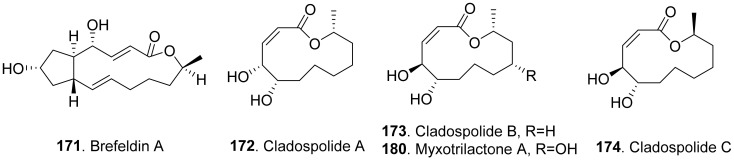

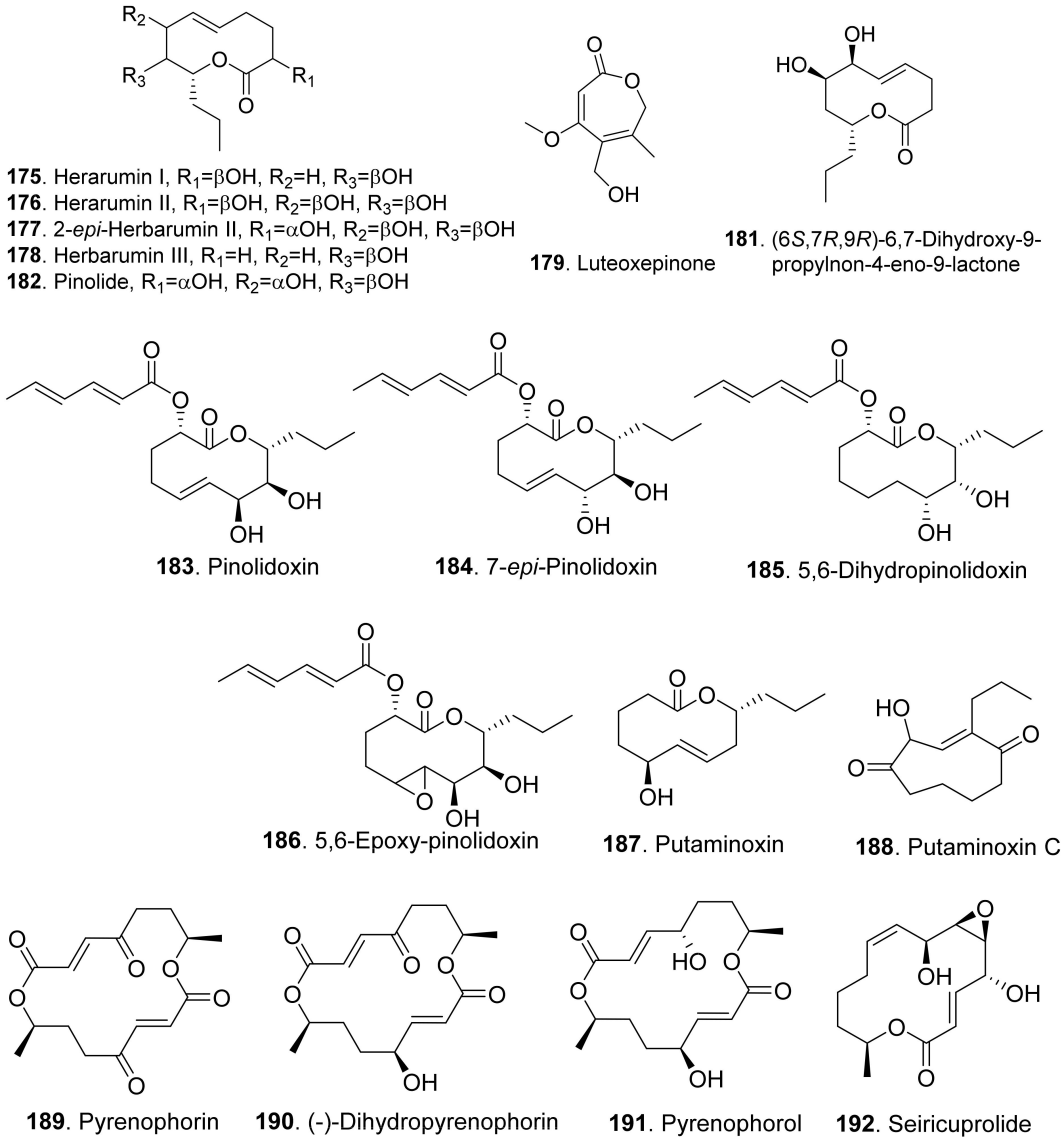
Structures of the phytotoxic aromatic-free macrolides isolated from fungi.

Phytotoxic aromatic-free macrolides from fungi with their structures are shown in Figure 18. Brefeldin A (**171**) was a bicyclic lactone isolated from the culture filtrates of *Alternaria zinnia*, which was used as the biocontrol agent of *Xanthium occidentale* (Compositae). Brefeldin A (**171**) was toxic to a series of the tested plants such as *Chenopodium album*, *Cirsium arvense*, *Mercurialis annua*, *Nicotiana tabacum*, *Sonchus oleraceus*, and *Xanthium occidentale* at 10^−4^ M by leaf pucture assay [101].

Cladospolides A (**172**) and B (**173**) were isomers isolated from the culture broth of *Cladosporium cladosporioides*. Cladospolide A (**172**) inhibited root elongation of lettuce and rice seedlings. However, cladospolide B (**173**) promoted root elongation of lettuce seedlings. It was interesting that these isomers had different plant growth regulatory activities [139]. Cladospolide C (**174**), a diastereomer of cladospolide A (**172**), was isolated from the culture filtrate of *Cladosporium tenuissimum*. Cladospolide C (**174**) inhibited shoot elongation of rice seedlings [140].

Cladospolide B (**173**) and myxotrilactone A (**180**) were isolated from the solid-substrate cultures of the endolichenic fungus *Myxotrichum* sp. Both compounds significantly inhibited shoot elongation of *Arabibopsis thaliana* by seedling growth assay [141].

Three nonenolides, namely herbarumins I (**175**), II (**176**), and III (**178**), were isolated from *Phoma herbarum*. These three compounds caused significant inhibition on radicle growth of *Amaranthus hypochondriacus* seedlings [142,143]. Both herbarumin II (**176**) and 2-*epi*-herbarumin II (**177**) were isolated from *Ascochyta pinodes* (syn. *Dymidella pinodes*), the pathogen of *Ascochyta* blight disease. These two compounds were toxic to *Pisum sativum* by leaf puncture assay [144].

Luteoxepinone (**179**) was isolated from *Neofusicoccum luteum*, the causal agent of Botryosphaeria dieback in Australia. It showed the phytotoxic effect by causing severe shriveling and withering on grapevine by leaf assay [37].

(6*S*,7*R*,9*R*)-6,7-Dihydroxy-9-propylnon-4-eno-9-lactone (**181**) was a phytotoxic nonenolide isolated from the solid cultures of endophytic fungus *Phomopsis* sp. HCCB03520 associated from *Achyranthes bidentata*. This metabolite showed phytotoxic activity on germination and radicle growth of *Medicago sativa*, *Trifolium hydridum*, and *Buchloe dactyloides* [145].

Pinolide (**182**) was isolated from *Ascochyta pinodes* (syn. *Dymidella pinodes*). This compound was toxic to *Pisum sativum* by leaf puncture assay [144].

Four nonenolide analogs called pinolidoxin (**183**), 7-*epi*-pinolidoxin (**184**), 5,6-dihydro-pinolidoxin (**185**), and 5,6-epoxy-pinolidoxin (**186**) were isolated from *Ascochyta pinodes*, the causal agent of anthracnose of pea (*Pisum sativum*). They caused necrotic lesions on pea and bean leaves tested by leaf puncture assay [146,147].

Putaminoxin (**187**) was isolated from the liquid culture filtrates of *Phoma putaminum*, the causal agent of leaf necrosis of *Erigeron annuus*. Putaminoxin (**187**) was toxic to a wide range of host and non-host plants with leaves of *E. annuus* being most sensitive [148]. Putaminoxin C (**188**) was isolated from the liquid culture filtrates of *Phoma putaminum*. This compound showed toxic effects similar to putamnoxin (**187**) [149].

Pyrenophorin (**189**) was isolated from the cultures of *Pyrenophora avenae*. It depressed radical growth of oat (*Avena sativa*) seedlings [150]. (−)-Dihydropyrenophorin (**190**) was isolated from the liquid culture of *Drechslera avenae*, the causal agent of leaf blotch of oats. This compound caused sunken lesions on oats and a variety of other plants at 3.2×10^−4^ M [151]. Pyrenophorol (**191**) was later isolated from *D. avenae* and was toxic to oats [152].

Seiricuprolide (**192**) was isolated from *Seiridium* sp., the pathogen causing canker disease of cypress. It showed minor inhibition to the test plants by cutting assay [153].

#### 2.2.5. Sorbicillinoids

**Figure 19:**
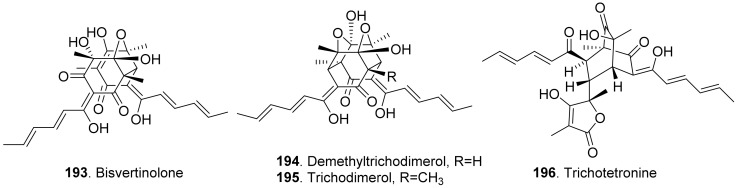
Structures of the phytotoxic sorbicillinoids isolated from fungi.

Sorbicillinoids (also called vertinoids) belong to hexaketide metabolites in which the cyclization has taken place on the carboxylate terminus. They have a variety of biological activities including cytotoxic, antioxidant, antiviral, antimicrobial and phytotoxic activity [154,155]. Four phytotoxic sorbicillinoids (Figure 19) named bisvertinolone (**193**), demethyltrichodimerol (**194**), trichodimerol (**195**), and trichotetronine (also called bislongiqinolide **196**) were isolated from the rice solid cultures of *Ustilaginoidea virens* (teleomorph: *Villosiclava virens*), the pathogen of rice false smut disease. These compounds were evaluated for their phytotoxic activity, and showed strong inhibition against the radicle and germ elongation of rice and lettuce seedlings. Among these compounds, bisvertinolone (**193**) displayed the strongest inhibition [156].

#### 2.2.6. Linear Polyketides

**Figure 20:**
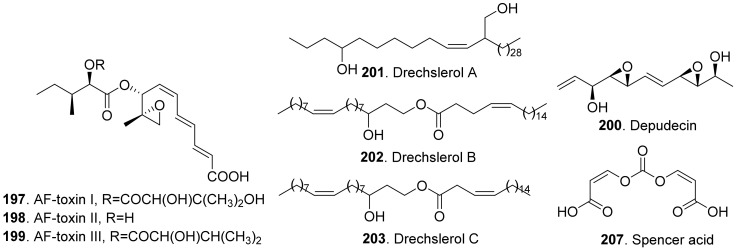

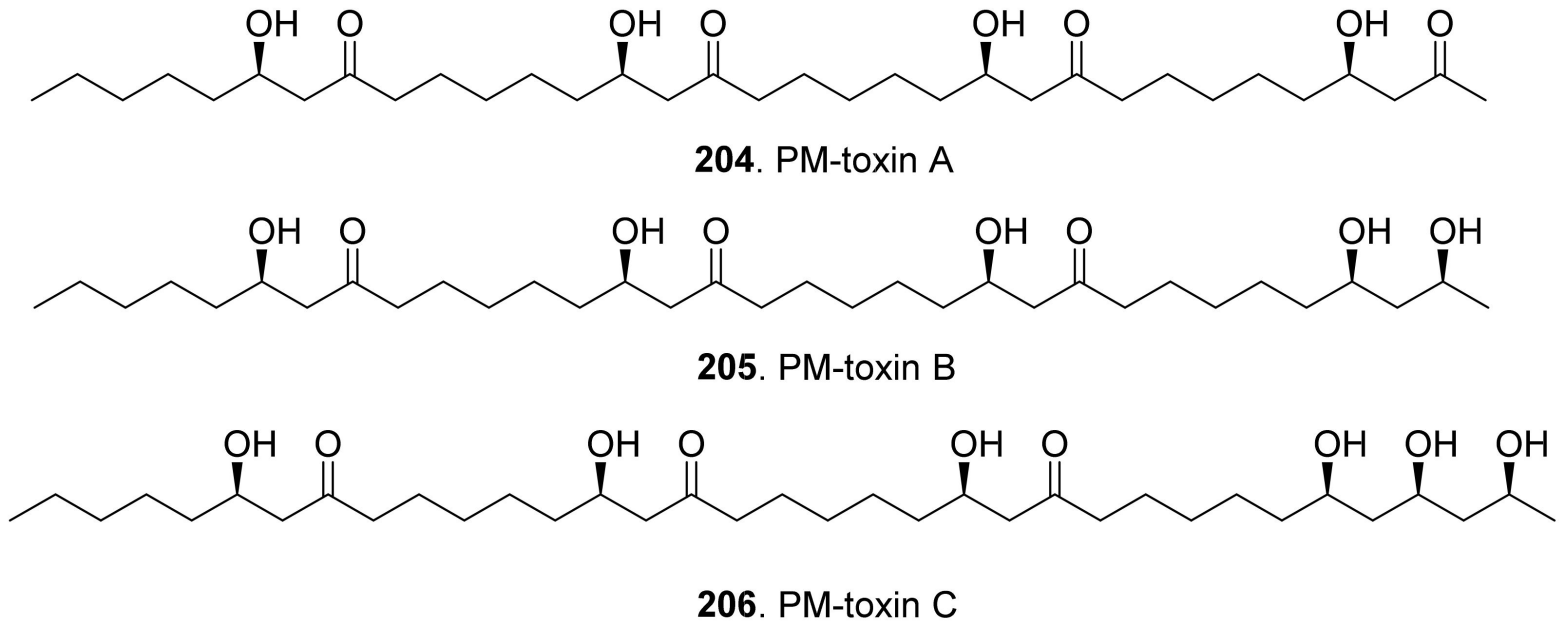
Structures of the phytotoxic linear polyketides isolated from fungi.

The structures of phytotoxic linear polyketides from fungi are shown in Figure 20. Three AF-toxins have been reported as AF-toxins I (**197**), II (**198**), and III (**199**), which were produced by *Alternaria alternata*, the pathogen of black spot of strawberry. They were host-specific toxins. AF-toxin I (**197**) also showed toxicity towards pear. AF toxin III (**199**) was highly toxic towards strawberry and less toxic to pear, while AF-toxin II (**198**) was toxic to pear [4,157].

Depudecin (**200**) was isolated from the weed pathogen *Nimbya scirpicola*. This metabolite produced necrotic lesions on kuroguwai, cowpea, and kidney bean by leaf-puncture assay, and inhibited the root elongation of lettuce seedlings. It did not show significant effects on the other test plants, which indicated that depudecin (**200**) was a host specific toxin [158].

Three host-specific toxins namely drechslerols A (**201**), B (**202**), and C (**203**) were successively isolated from the culture filtrate of *Drechslera maydis*, the pathogen of leaf blight disease of *Costus speciosus*. They all caused necrotic and chlorotic lessions on the leaves of *C. speciosus*, and inhibited root growth of wheat seedlings [159,160,161].

Three host-specific toxins namely PM-toxins A (**204**), B (**205**), and C (**206**) were isolated from the corn pathogen *Phyllosticta maydis*. They belonged to the linear polyketides with phytotoxicity toward the tissues and mitochondria obtained from susceptible corn varieties [162].

Spencer acid (**207**) was a diacrylic acid derivative isolated from *Spencermartinsia viticola*, the causal agent of Botryosphaeria dieback on grapevine in Australia. It exhibited strong phytotoxicity on *Vitis lambrausca* and *V. vinifera* cv. Shiraz by grapevine leaf assay [163].

## 3. Phenols and Phenolic Acids

**Figure 21:**
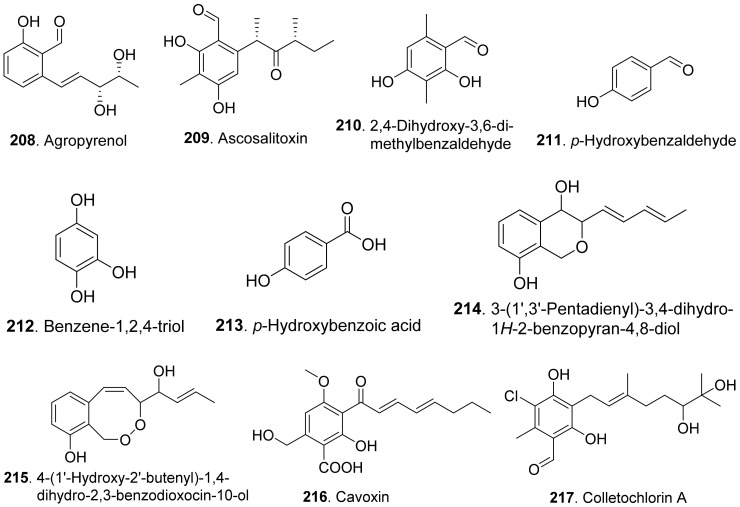

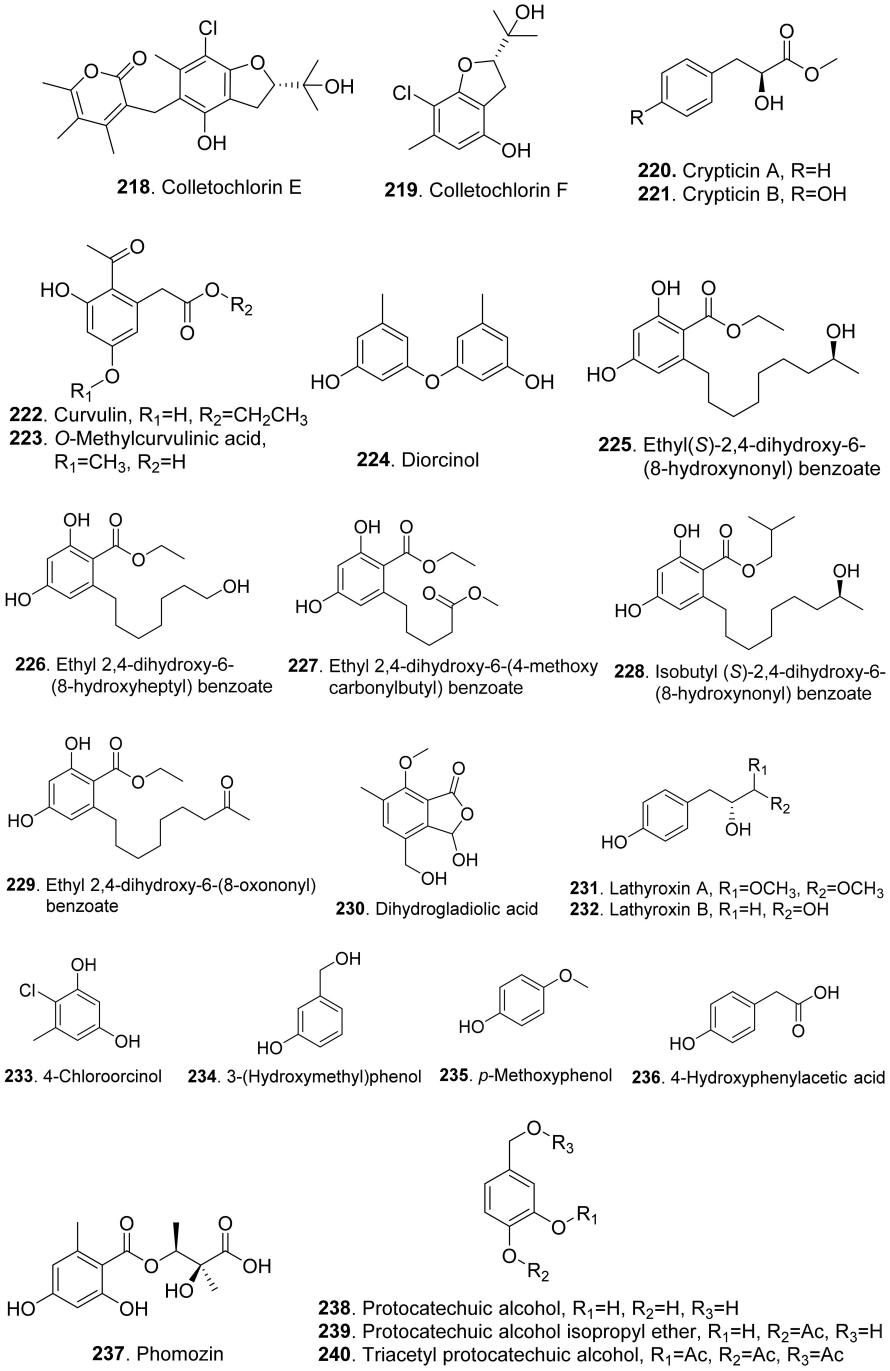

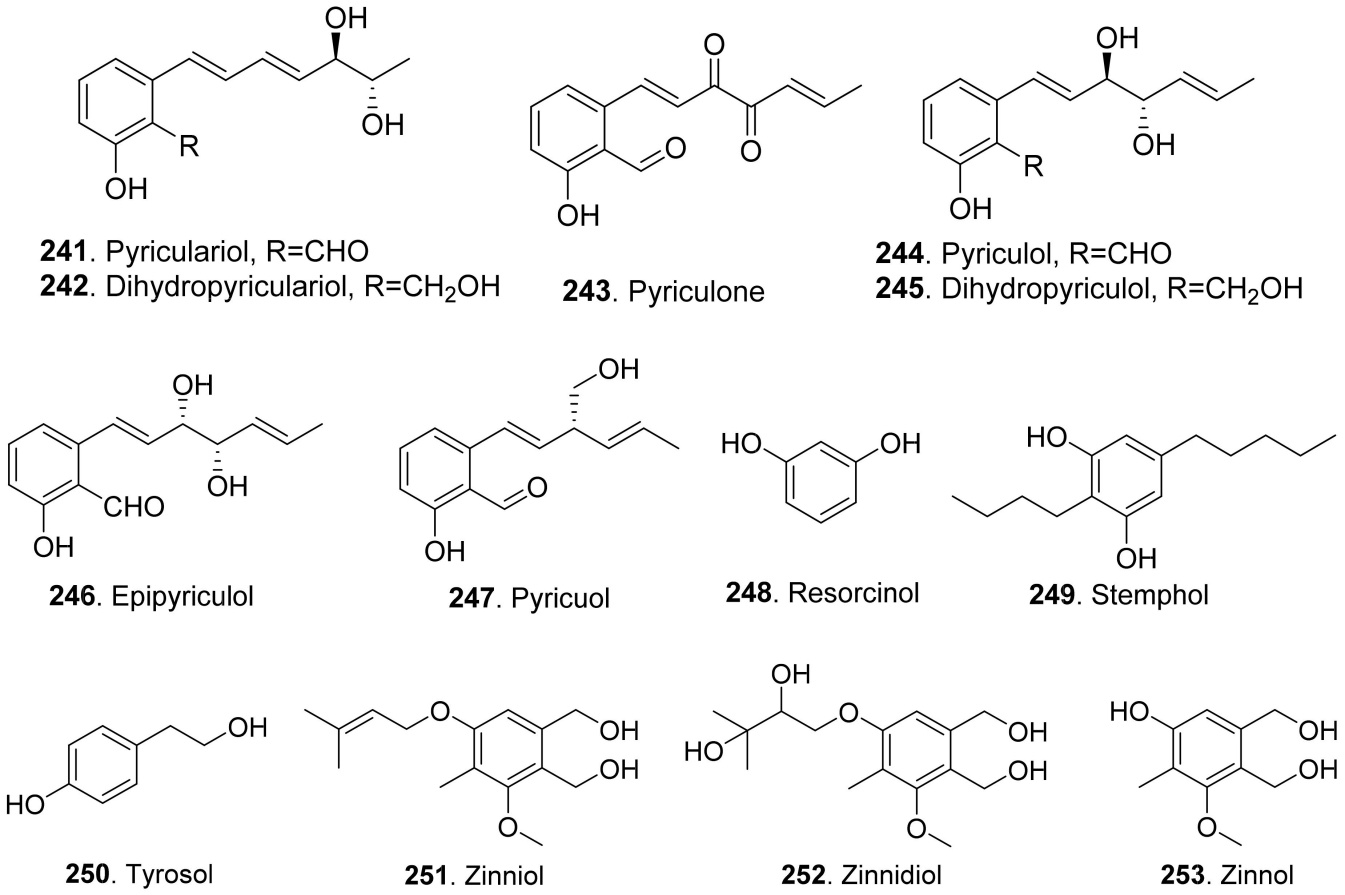
Structures of the phytotoxic phenols and phenolic acids isolated from fungi.

Phenols and phenolic acids are mixed biosynthetic origins. Most phenol and phenolc acid derivatives are of polyketide origin such as salicylaldehyde analogues. Other biosynthetic origins include shikimic acid and mevalonic acid pathways [164]. The structures of phytotoxic phenols and phenolic acids from fungi are shown in Figure 21.

Agropyrenol (**208**) was a dihydroxypentenyl substituted salicyladehyde isolated from the liquid cultures of *Ascochyta agropyrina* var. *nana*. When the leaves of several weed plants (i.e., *Mercurialis annua*, *Chenopodium album,* and *Setaria viridis*) were assayed, agropyrenol (**208**) was proved to be phytotoxic to cause the appearance of necrotic lesions by leaf puncture assay [57].

Ascosalitoxin (**209**) was a trisubstituted salicylic aldehyde which belonged to the methylated hexaketide via polyketide biosynthetic pathway [165]. This metabolite was isolated from *Ascochyta pisi* var. *pisi* to show phytotoxic activities on the leaves and pods of pea and bean, as well as on tomato seedlings [166].

Moreover, 2,4-dihydroxy-3,6-dimethylbenzaldehyde (**210**) isolated from *Leptosphaeria maculans* was virulent on canola. This metabolite had strong root and hypocotyl growth inhibition on lettuce seedlings [167].

*p*-Hydroxybenzaldehyde (**211**) was isolated from *Ascochyta lentis* var. *lathyri*, the pathogen of grapevine trunk. This compound caused necrosis on leaves of seven plant species by leaf puncture assay, and inhibited seed germination and rootlet elongation of *Phelipanche ramose* [18]

Six phenols, namely benzene-1,2,4-triol (**212**), 3-(hydroxymethyl)phenol (**234**), protocatechuic alcohol (**238**), protocatechuic alcohol isopropyl ether (**239**), triacetyl protocatechuic alcohol (**240**), and resorcinol (**248**), were isolated from *Dothiorella viidmadera*, the causal agent of grapevine trunk desease. They all showed phytotoxicity to tomato and grapevine. Among them, resorcinol (**248**) was the most phytotoxic compound on grapevine leaves by causing severe shriveling of the leaves [168].

*p*-Hydroxybenzoic acid (**213**) was isolated from *Alternaria dauci*, which was the causal agent of *Alternaria* leaf blight. It showed an important phytotoxic activity when tested in the leaf-spot assay on parsley (*Petroselinum crispum*), in the leaf infiltration assay on tobacco (*Nicotiana alata*) and marigold (*Tagetes erecta*), and in the immersion assay on parsley and parsnip (*Pastinaca sativa*) leaves. It might play an important role in the pathogenicity of the fungus [169].

Two phenolic acids namely *p*-hydroxybenzoic acid (**213**) and 4-hydroxyphenylacetic acid (**236**) were isolated from *Spencermartinsia viticola*, the causal agent of Botryosphaeria dieback on grapevine in Australia. Both metabolites showed strong phytotoxicity on *Vitis lambrausca* and *Vitis vinifera* cv. Shiraz by grapevine leaf assay [163].

Two pyriculol-related phytotoxins named 3-(1’,3’-pentadienyl)-3,4-dihydro-l*H*-2-benzopyran-4,8-diol (**214**) and 4-(1’-hydroxy-2’-butenyl)-1,4-dihydro-2,3-benzodioxocin-10-ol (**215**) were isolated from solid cultures of *Pyricularia oryzae*. Both compounds gave a yellowish-orange symptom to rice leaves by leaf puncture assay [170].

Cavoxin (**216**) was a tetrasubstituted benzoic acid derivative isolated from *Phoma cava*, the pathogen of chestnut (*Castanea* spp.). Cavoxin (**216**) caused vascular browning and rapid wilting of the leaflets by tomato cutting assay [171].

Colletochlorins A (**217**), E (**218**), and F (**219**) were isolated from *Colletotrichum higginsianum*. Colletochlorin A (**217**) was a chlorinated 3-diprenyl orsellinaldehyde derivative. Colletochlorin F (**219**) was a dihydrobenzofuran. Colletochlorin E (**218**) was considered to be the combination of a tetrasubstituted α-pyranone with colletochlorin F (**219**). Among three compounds, collethochlorin F (**219**) caused the fastest appearance of quite large necrosis on punctured *Sonchus arvensis* leaves. Tomato punctured leaves were less sensitive to these compounds by comparing with *S. arvensis* leaves [172].

Crypticins A (**220**) and B (**221**) were isolated from the culture filtrates of *Diaporthella cryptica*, the emerging hazelnut pathogen [173].

Curvulin (**222**) and *O*-methylcurvulinic acid (**223**) were isolated from *Drechslera indica* which caused leaf necroses on purslane (*Portulaca oleracea*) and spiny amaranth (*Amaranthus spinosus*). Curvulin (**222**) was only toxic to the host plants purslane and spiny amaranth. *O*-Methylcurvulinic acid (**223**) was also toxic to the other test plant species besides host plants [174].

Diorcinol (or called 3,3′-dihydroxy-5,5′-dimethyldiphenyl ether, **224**) was isolated from *Diplodia corticola*, an oak pathogen. This metabolite was toxic to the leaves of *Quercus afares*, *Q. suber*, *Q. ilex* and *Celtis australis* at 1 mg/mL by causing necrotic lesions [175]. Diorcinol (**224**) was also isolated from the endophytic fungus *Epichloe bromicola* obtained from *Elymus tangutorum* grass. It displayed obvious inhibition on the root and shoot growth of *Lolium perenne* and *Poa crymophila* seedlings, and was as active as the positive control glyphosate [176].

Five β-resorcylic acid derivatives namely ethyl (*S*)-2,4-dihydroxy-6-(8-hydroxynonyl) benzoate (**225**), ethyl 2,4-dihydroxy-6-(8-hydroxyheptyl) benzoate (**226**), ethyl 2,4-dihydroxy-6-(4-methoxycarbonylbutyl) benzoate (**227**), isobutyl (*S*)-2,4-dihydroxy-6-(8-hydroxynonyl) benzoate (**228**), and ethyl 2,4-dihydroxy-6-(8-oxononyl) benzoate (**229**) were isolated from rice fermentation cultures of the endophytic fungus *Lasiodiplodia theobromae* derived from the mangrove plant *Xylocarpus granatum*. These metabolites all stimulated root elongation of Luctuca sativa seedlings. In addition, ethyl 2,4-dihydroxy-6-(4-methoxycarbonylbutyl) benzoate (**227**) inhibited root elongation of *Digitaria ciliaris* [104].

Dihydrogladiolic acid (**230**) was a tetrasubstitued benzofuranone isolated from *Phoma asparagi*. It showed inhibitory activity on root elongation of lettuce seedlings [177].

Lathyroxins A (**231**) and B (**232**) were two phytotoxic *p*-hydroxyphenylpropanoids isolated from Lathyroxin A (**231**) caused necrosis on leaves of *Lupinis albus* and *Sonchus oleraceus*. Lathyroxin B (**232**) caused clear necrosis on leaves of all the tested plants including *Sonchus oleraceus*, *Lycopersicon esculentum*, *Phaseolus vulgaris,* and *Lens culinaris*. Both compounds inhibited seed germination and rootlet elongation of the parasitic weed *Phelipanche ramosa* [178].

4-Chloroorcinol (**233**) was isolated *Colletotrichum higginsianum*. This compound caused necrosis on the punctured leaves of *Sonchus arvensis* and tomato leaves [172].

*p*-Methoxyphenol (**235**) was isolated from the culture filtrates of *Ascochyta lentis* var. *lathyri*, the causal agent of *Ascochyta* blight of grass pea (*Lathyrus sativus*). *p*-Methoxyphenol (**235**) caused clear necrosis on leaves of seven test plants, and inhibited seed germination and rootlet elongation of the parasitic weed *Phelipanche ramosa* [178].

Phomozin (**237**) was an ester of orsellinic acid and dimethylglyceric acid. It was isolated from *Phomopsis helianthi* which was the causal agent of leaf necrosis and steam cankers of sunflowers. Phomozin (**237**) was thought as a host-specific phytotoxin by leaf puncture assay and cutting test [179].

Pyriculol (**244**) derivatives phytotoxins were mainly isolated from rice blast pathogen *Magnaporthe oryzae* (anamorph: *Pyricularia oryzae*) [180,181]. They are classified into two groups [181]: alcohol-type such as dihydropyriculariol (**242**) and dihydropyriculol (**245**), and aldehyde-type, such as pyriculariol (**241**) and pyriculol (**244**). Pyricuol (**247**) exhibited stronger phytotoxic activity toward rice than pyriculol (**244**) and dihydropyriculol (**245**) [182]. It has been shown that aldehyde derivatives induced lesion-like necrosis on rice leaves, while alcohol derivatives were inactive [183]. Epipyriculol (**246**) was isolated from the cultures of *Pyricularia oryzae*. It was the (10*S*)-isomer of pyriculol (**244**). Both pyriculol (**244**) and epipyriculol (**246**) caused a similar brownish symptom on the rice leaves by leaf puncture assay [170]. Dihydroxypyriculol (**245**) from *Pyricularia grisea*, the pathogen of buffelgrass (*Cenchrus ciliaris*), showed a significant stimulating effect of radical elongation of buffelgrass by seedling growth assay [32].

Stemphol (**249**) was isolated from *Stemphylium botryosum*, the pathogen of oilseed rape. This metabolite was toxic to the cells of oilseed rape and chickpea by using cell viability assay [184].

Phytotoxic tyrosol (also called 2-(4-hydroxyphenyl)-ethanol, **250**) was isolated from a series of fungi such as *Ascochyta lentis* [87], *Ascochyta lentis* var. *lathyri* [178], *Diaporthe eres* [31], and *Stilbocrea macrostoma* [108].

Three phenol derivatives namely zinniol (**251**), zinnidiol (**252**) and zinnol (**253**) were isolated from the fungus *Alternaria cichorii*, the pathogen of foliar blight disease of Russian knapweed (*Acroptilon repens*). They were toxic to the leaves of Russian knapweed by in vitro leaf puncture assay [185]. Zinniol (**251**), which was also isolated from *Alternaria solani*, inhibited seedling growth of tomato [120].

## 4. Terpenoids

The phytotoxic terpenoids from fungi include monoterpenoids, sesquiterpenoids, diterpenoids, sesterterpenoids, triterpenoids, and meroterpenoids.

### 4.1. Monoterpenoids

**Figure 22:**
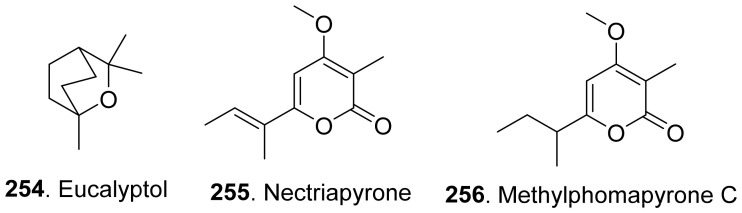
Structures of the phytotoxic monoterpenoids isolated from fungi.

Phytotoxic monoterpenoids from fungi with their structures are shown in Figure 22. The volatile and semi-volatile organic compounds with phytotoxic and antimicrobial activities were isolated from the endophytic fungus *Hypoxy anthochroum* strain Blaci isolated from *Bursera lancifolia* (Burseaceae). Eucalyptol (**254**), the main constituent among the volatile organic compounds, showed the highest phytotoxic effect on seed germination, root elongation and seedling respiration of *Amaranthus hypochondriacus*, *Panicum miliaceum*, *Trifolium pratense,* and *Medicago sativa* [186].

Nectriapyrone (**255**) was a monoterpenoid α-pyrone from the pathogen *Phomopsis foeniculi* (teleomorph: *Diaporthe angelicae*) of fennel (*Foeniculum vulgare*). Nectriapyrone (**255**) showed a modulated phytotoxicity on the detached tomato leaves. Methylphomapyrone C (**256**) was the dihydroderivative of nectriapyrone (**255**) [75]. Both nectriapyrone (**255**) and methylphomapyrone C (**256**) were toxic to a number of non-host plants *Cirsium arvense*, *Sonchus oleraceus,* and *Chenopodium album* with a leaf puncture assay [127].

### 4.2. Sesquiterpenoids

**Figure 23:**
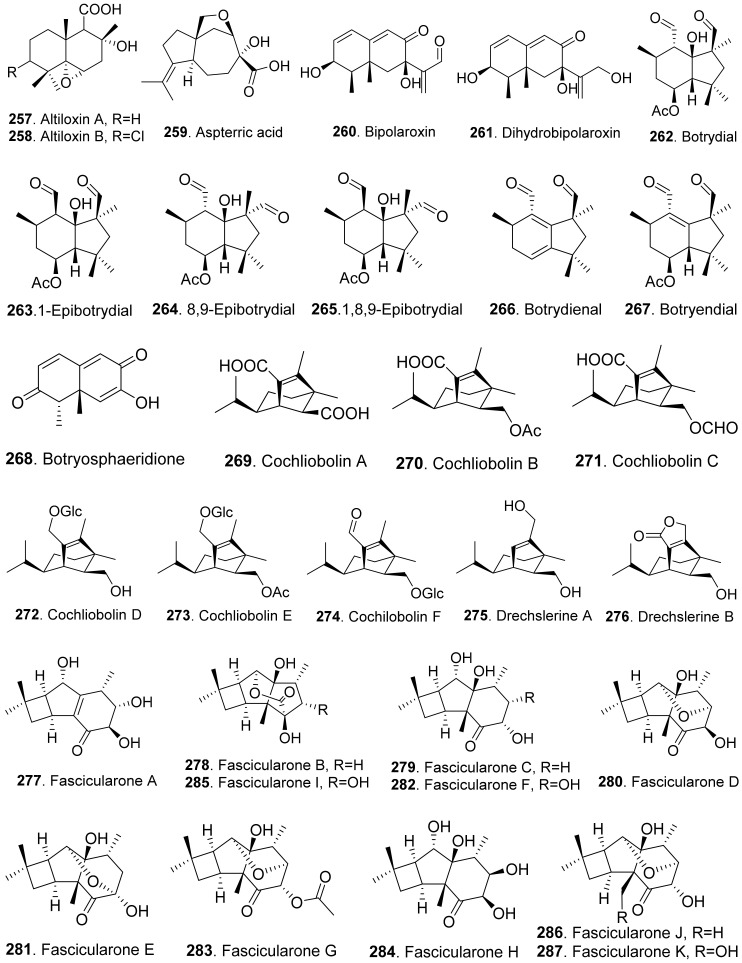

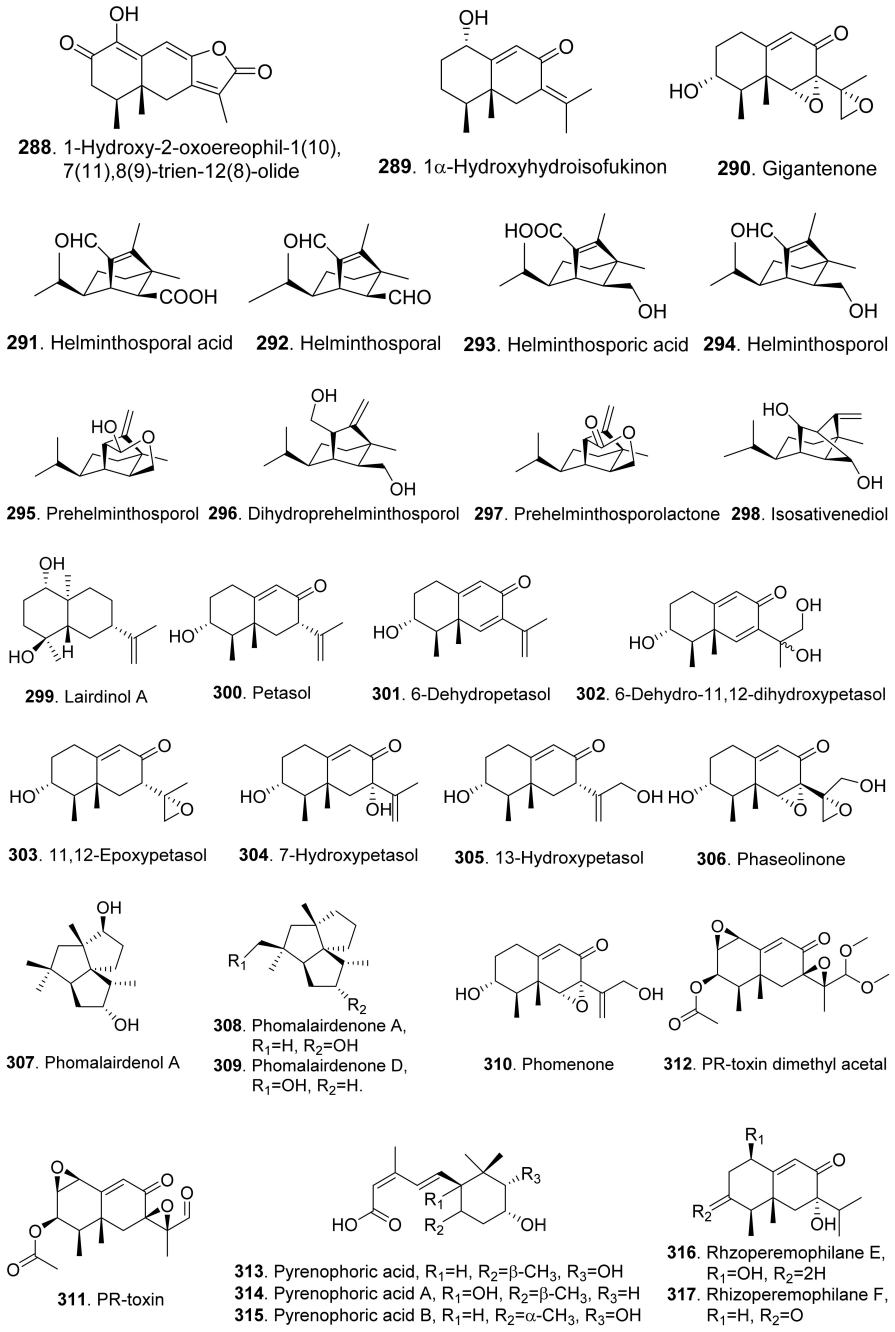

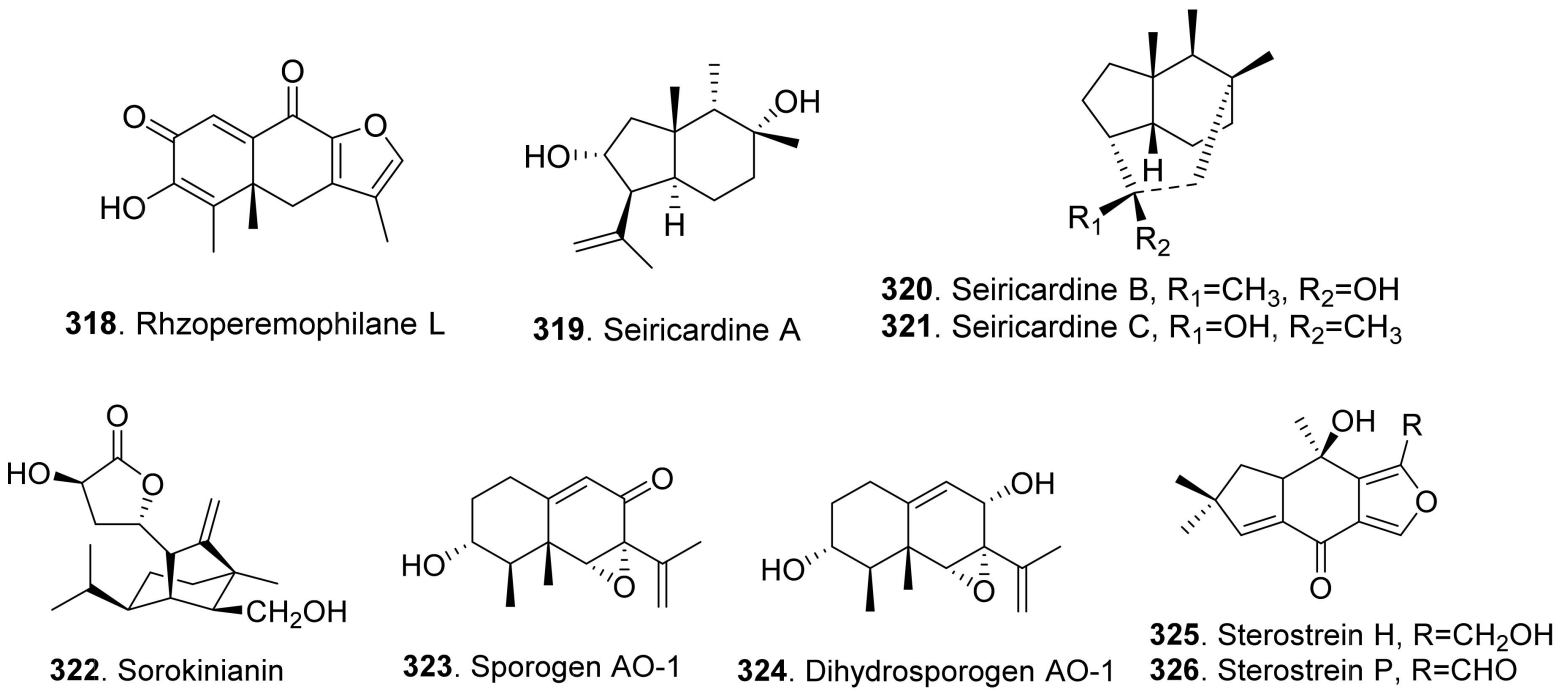
Structures of the phytotoxic sesquiterpenoids isolated from fungi.

Many sesquiterpenoids from fungi showed phytotoxic activities. Their structures are shown in Figure 23. Two drimane-type sesquiterpenoids, named altiloxins A (**257**) and B (**258**), were isolated as the main phytotoxins from *Phoma asparagi*, the causal agent of stem blight disease on saparagus. When tested on root elongation of the non-host lettuce seedlings, both compounds showed a weak inhibitory activity. Meanwhile, in the same assay carried out on the host plant at 10 μg/mL, they inhibited the root elongation of 48.2% and 48.5%, respectively [187].

Aspterric acid (**259**) was previously found from *Aspergillus terreus* to inhibit the pollen development of *Arabidopsis thaliana*. However, the mode of action was not clear [188]. This compound was later found to inhibit dihydroxy acid dehydratase (DHAD), which is an essential and highly conserved enzyme among plant species that catalyses β-dehydration reactions to yield α-keto acid precursors to isoleucine, valine and leucine. DHAD along with other two enzymes: acetolactate synthase (ALS) and actohydroxy acid isomeroreductase (KARI) are three enzymes in the plant branched-chain amino acid (BCAA) biosynthetic pathway, which is essential for plant growth [189].

Bipolaroxin (**260**) and dihydrobipolaroxin (**261**) were isolated from *Bipolaris cynodontis*, the pathogen of Bermuda grass (*Cynodon dactylon*). Bipolaroxin (**260**) displayed selective phytotoxicity. Dihydrobipolaroxin (**261**) showed no phytotoxicity at 3.8 mM against Bermuda grass, goosegrass, wheat and barley, which indicated that the C12 aldehyde was essential for activity [190]. Bipolaroxin (**260**) was also isolated from *Bipolaris sorokiniana*, the causal agent of spot blotch of wheat. Wheat, barley, maize, and sorghum were sensitive to bipolaroxin (**260**) at 30 ng/mL while chickpea, tomato, cotton, and rice were insensitive [191].

Botrydial (**262**) and its epimer metabolites 1-epibotrydial (**263**), 8,9-epibotrydial (**264**) and 1,8,9-epibtrotrydial (**265**), as well as its unsaturated dialdehydes botrydienal (**266**) and botryendial (**267**) were isolated from the phytopathogen *Botrytis cinerea*. The unsaturated botrydienal (**266**) and botryendial (**267**) were more phytotoxic than epimers 1-epibotrydial (**263**), 8,9-epibotrydial (**264**) and 1,8,9-epibtrotrydial (**265**) by tabacco leaf assay [192]. Botrydial (**262**) from *Botrytis cinerea* was found to trigger phosphatidic acid production in tomato suspension cells, and phosphatidic acid was found to positively regulate the production of reactive oxygen species (ROS) [193].

Botryosphaeridione (**268**) was a trinor-eremophilane sesquiterpene isolated from *Phoma* sp. LN-16, an endophytic fungus associated with *Melia azedarach*. It inhibited the seed germination of lettuce (*Lactuca sativa*) with IC_50_ as 93.64 μg/mL [194].

A series of *seco*-sativene sesquiterpenoids including cochliobolins A-F (**269**-**274**), drechslerines A (**275**) and B (**276**), helminthosporal acid (**291**), helminthosporic acid (**293**), helminthosporol (**294**), and isosativenediol (**298**) were isolated from *Cochiliobolus sativus*, the endophytic fungus isolated from a desert plant *Artemisia desertorum*. Among them, cochliobolin F (**274**), drechslerine B (**276**), helminthosporal acid (**291**), and helminthosporic acid (**293**) displayed strong phytotoxic effects on corn leaves by producing visible lesions [23]. Both helminthosporal acid (**291**) and helminthosporol (**294**) were also isolated from *Bipolaris sorokiniana*. These two compounds inhibited seed germination of lettuce [195]. Helminthosporal (**292**) isolated from *Helminthosporium sativum* was toxic to barley root explants by seedling growth assay. It reacted directly with both the plasmalemma and the tonoplast membranes of the beet root cells [196].

Eleven sesquiterpenoids namely fascularones A-K (**277**-**287**) were isolated from the culture broth of *Naematoloma fasciculare*, a bitter poisonous mushroom distributed in northeast Japan. They all contained a *cis*-fused four-membered ring moiety, and promoted radicle elongation of lettuce seedlings [197,198,199,200].

In addition, 1-hydroxy-2-oxoeremophil-1(10),7(11),8(9)-trien-12(8)-olide (**288**) from *Malbranchea aurantiaca* showed significant inhibition of radicle growth of *Amaranthus hypochondriacus* seedlings with IC_50_ value of 6.57 μM. In addition, this compound inhibited activation of the calmodulin-dependent enzyme cAMP phosphodiesterase with IC_50_ of 10.2 μM [111].

Prehelminthosporol (**295**) was isolated from *Dreschlera sorokiana* (syn. *Helminthosprorium sativum*, *Bipolaris sorokiniana*). This metabolite was a plant growth regulator that promoted shoot growth of rice seedlings but inhibited the coleoptile growth of wheat seedlings [201]. Prehelminthosporol (**295**) and dihydroprehelminthosporol (**296**) were isolated from the culture filtrates of *Bipolaris* species which was the pathogen of Johnson grass (*Sorghum halepense*), one of the worst weeds in tropical and subtropical areas of the world. Both metabolites were toxic towards sorghum (*Sorghum bicolor*) in leaf spot assay [202]. Prehelminthosporolactone (**297**) was latter isolated from the the culture filtrates of *Bipolaris* species to show toxic to the leaves of sorghum and sicklepod (*Cassia obtusifolia*) [203].

Nine phytotoxic eremophilane-type sesquiterpenoids namely gigantenone (**290**), petasol (**300**), 6-dehydropetasol (**301**), 6-dehydro-11,12-dihydroxypetasol (**302**), 11,12-epoxypetasol (**303**), 7-hyroxypetasol (**304**), 13-hydroxypetasol (**305**), phaseolinone (**306**), and phomenone (**310**) were isolated from some fungi such as *Drechslera gigantean* and *Macrophomina phaseolina*. They caused necrotic lesions on several grasses such as crabgrass (*Digitaria* spp.), quackgrass (*Agropyron repens*), and Bermuda grass (*Cynodon dactylon*) by leaf puncture assay [204]. Phaseolinone (**306**) also caused non-specific leaf necrosis on several plants, and inhibited seed germination of soybean [205].

Lairdionol A (**299**), phomalairdenol A (**307**), and phomalairdenones A (**308**) and D (**309**), were isolated from *Leptosphaeria maculans*. They showed selective phytotoxicity to brown mustard [206,207].

Two eremophilane-type sesquiterpenoids namely phomenone (**310**) and PR-toxin (**311**) were isolated from the cultures of *Phoma destructiva*. They inhibited radical elongation and shoot growth of tomato seedlings at 10^−4^ M. [208].

Pyrenophoric acid (**313**) and pyrenophoric acids B (**314**) and C (**315**) were isolated from seed pathogen *Pyrenophora semeniperda* of cheatgrass (*Bromus tectorum*). Three metabolites showed phytotoxic activity by reducing coleoptile elongation of cheatgrass seedlings [209,210]. Among three metabolites, pyrenophoric acid B (**314**) was the most phytotoxic to use the abscisic acid (ABA) biosynthesis pathway at the level of alcohol dehydrogenase ABA2 to reduce seed germination of cheatgrass [211].

Moreover, 1α-hydroxyhydroisofukinon (**289**), PR-toxin dimethyl acetal (**312**), and rhizoperemophilanes E (**316**), F (**317**), and L (**318**) were nitrogen-containing eremophilane-type sesquiterpenoids isolated from the endophytic fungus *Rhizopycnis vagum*. These metabolites inhibited radicle elongation of rice seedlings [212].

Seiricardines A (**319**), B (**320**), and C (**321**) were separately isolated from the culture filtrates of *Seiridium cardinale*, *S. cupressi*, and *S. unicorne*, that all were associated with canker disease of cypress (*Cupressus sempervirens*) in the Mediterranean area [213,214]. The solution of seiricardine A (**319**) at 0.3 mg/mL was absorbed by severed twigs of cypress to cause the leaf yellowing and browning. Subperidermal injection of the solution of seiricardine A (**319**) at 0.1 mg/mL into young cypress trees caused necrotic lesions on the stem and a diffuse yellowing of adjacent twigs [213]. Seiricardines B (**320**) and C (**321**) were epimeric diastereomers. They showed similar phytotoxic activity to sericardine A (**319**) [214].

Sorokinianin (**322**) was isolated from the culture broth of *Bipolaris sorokiniana*, the pathogen of barley. This compound inhibited germination of the seeds of barley (*Hordeum vulgare*) [215].

Two eremophilane-type sesquiterpenes, namely sporogen AO-1 (also called 13-desoxyphomenone, **323**) and dihydrosporogen AO-1 (**324**), were isolated from the coprophilous fungus *Penicillium* sp. G1-a14. Both sporogen AO-1 (**323**) and dihydrosporogen AO-1 (**324**) caused significant inhibition of radicle growth against *Amaranthus hypochondriacus* (IC_50_ value of 0.17 mM for both compounds) and *Echinochloa crus-galli* (IC_50_ values of 0.17 mM and 0.30 mM, respectively) [216]. Sporogen AO-1 (**323**) isolated from *Hansfordia* sp. 185-94 showed inhibition on seed germination of *Lepidium sativum* and *Setaria italic* [217].

Sterostreins H (**325**) and P (**326**) from *Stereum complicatum* showed inhibition on seed germination and growth of lettuce (*Lactuca sativa*), bentgrass (*Agrostis stolonifera*), and *Lemna paucicostata* [218].

### 4.3. Diterpenoids

**Figure 24:**
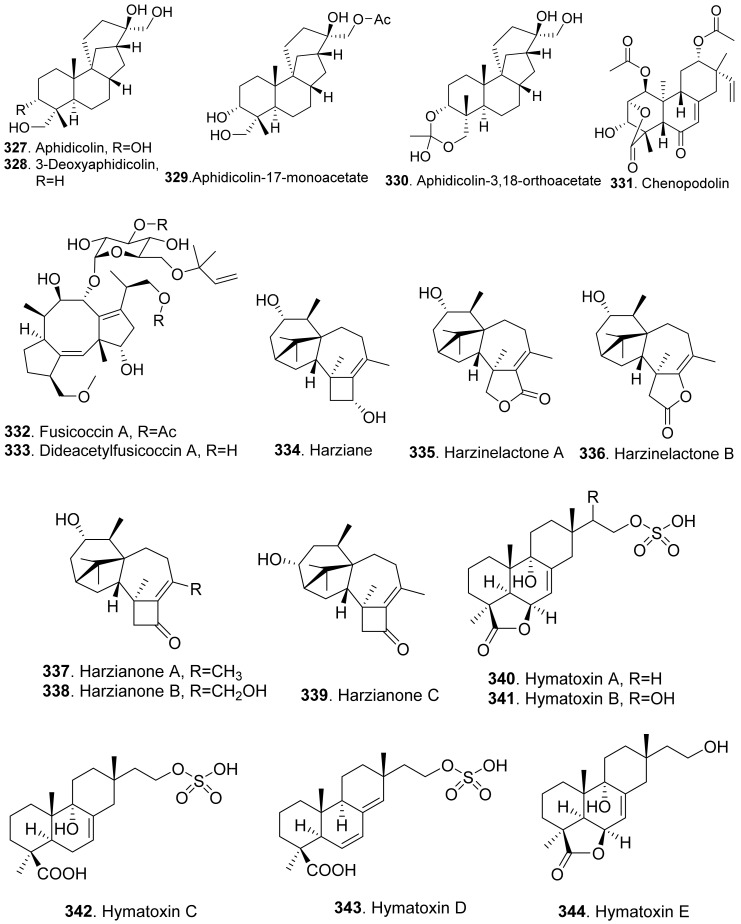

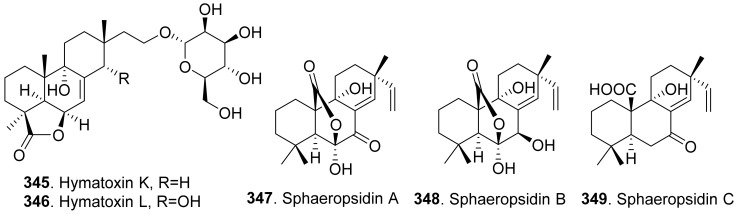
Structures of the phytotoxic diterpenoids isolated from fungi.

Many diterpenoids from fungi exhibit phytotoxic activities. Structures of phytotoxic diterpenoids from fungi are shown in Figure 24. Aphidicolin (**327**) and its congeners 3-deoxyaphidicolin (**328**), aphidicolin-17-monoacetate (**329**), and aphidicolin-3,18-orthoacetate (**330**) were isolated from *Phoma betae*, the pathogen of leaf spot disease on sugar beet. When these compounds were tested at 10^−4^ M, their inhibitory rates on root growth of lettuce seedlings were 74.3%, 50.9%, 58.1%, and 54.5%, respectively [219].

Chenopodolin (**331**) was an unrearranged *ent*-pimaradiene diterpene isolated from the pathogen *Phoma chenopodiicola*, which was proposed for the biological control of *Chenopodium album*, a common worldwide weed of arable crops such as sugar beet and maize. At concentration of 2 mg/mL, the compound caused necrotic lesions on the leaves of *Mercurialis annua*, *Cirsium arvense*, and *Setaria viride* [220].

Fusicoccn A (**332**) and dideacetylfusicoccin A (**333**) were diterpene glycosides produced by the plant pathogenic fungus *Fusicoccum amygdali* (syn. *Phomopsis amygdali*) with a unque *O*-prenylated glucose moiety. They stimulated seed germination of the parasitic weeds *Orobanche* spp. [221]. Further mechanism investigation showed that fusicoccn A (**332**) binded to a hydrophobic cavity in plant 14-3-3 proteins and stabilized the interaction with the C-terminal phosphorylated domain of plasma membrane H^+^-ATPase, thereby promoting stomatal opening and eventually leading to plant death [222].

Six diterpenoids named harziane (**334**), harzianelactones A (**335**) and B (**336**), and harzianones A (**337**), B (**338**) and C (**339**) were isolated from the soft coral-derived fungus *Trichoderma harzianum* XS-20090075. These harziane diterpenoids exhibited potent phytotoxicity against seedling growth of amaranth (*Amaranthus retroflexus*) and lettuce (*Lactuca sativa*) [223].

Some phytotoxic pimarane diterpenoids were isolated from the fungus *Hypoxylon mammatum*, the stem canker pathogen of aspen (*Populus* spp.). Hymatoxin A (**340**) was isolated from the culture broth of pathogenic fungus *H. mammatum* [69,224]. Hymatoxins B-E (**341**-**344**) [69], and hymatoxins K (**345**) and L (**346**) [225] were further isolated from the culture broth of *Hypoxylon mammatum*. Hamatoxins A-D (**340**-**343**) each had a sulfate group, and hamatoxins K (**345**) and L (**346**) were mannopyranosides, which made them hydrosoluble. These pimarane diterpenoids were all phytotoxic to the leaves of aspen.

Sphaeropsidin A (**347**) was a pimarane diterpene from *Sphaeropsis sapinea* f.sp. *cupressi*, the pathogen of a canker disease of cypress (*Cupressus sempervirens*). When this compound was absorbed by the servered twigs of *Cupressus* and cuttings of two herbaceous plants (tomato and oat), sphaeropsidin A (**347**) at 0.1 mg/mL produced leaf yellowing, browning and dieback [226]. The pathogen *Sphaeropsis sapinea* f.sp. *cupressi* also produced phytotoxins sphaeropsidins B (**348**) and C (**349**), and another pathogen *Diplodia mutila* produced phytotoxins sphaeropsidins A (**347**) and B (**348**) [227].

### 4.4. Sesterterpenoids

**Figure 25:**
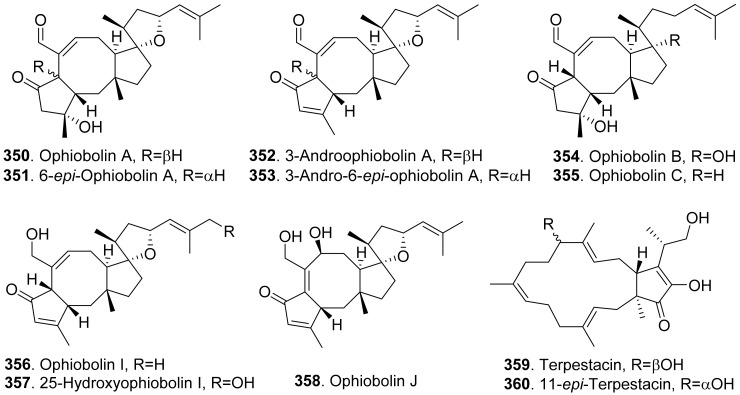
Structures of the phytotoxic sesterterpenoids isolated from fungi.

Fungal phytotoxic sesterterpenoids from fungi with their structures are shown in Figure 25. Representative phytotoxic sesterterpenoids are ophiobolin congeners which structures contain a tricyclic 5-8-5 carbotricyclic skeleton. Ophiobolins and their biological activities were well reviewed [228,229,230]. *Bipolaris oryzae* (syn. *Helminthosporium oryzae*, *Cochliobolus oryzae*, and *Drechslera oryzae*) is the pathogen of rice, maize and sorghum. It can produce phytotoxic ophiobolins.

Four sesterterpenoids namely ophiobolin A (**350**), 6-*epi*-ophiobolin A (**351**), 3-anhydroophiobolin A (**352**), and 3-anhydro-6-epi-ophiobolin A (**353**) were produced by *Bipolaris oryzae*. They were toxic to the photosynthesis of rice plants by measurement of spinach leaf photosynthesis [231].

Ophiobolin A (**350**), 6-*epi*-ophiobolin A (**351**), 3-anhydro-6-*epi*-ophiobolin A (**353**), ophiobolin B (**354**), ophiobolin I (**356**), and ophiobolin J (**358**) were isolated from the liquid cultures of *Drechslera gigantean*, a cosmopolitan fungal pathogen of plants. They were toxic to large crabgrass (*Digitaria sanguinalis*) by leaf puncture detached assay. These ophiobolin sesterterpenoids were considered as potential natural herbicides [232].

Ophiobolin A (**350**), 6-*epi*-ophiobolin A (**351**), 3-anhydroophiobolin A (**352**), and 3-anhydro-6-*epi*-ophiobolin A (**353**) were also isolated from plant pathogen *Bipolaris sorghicola*. When tested on several plants by using a leaf spot assay, ophiobolin A (**350**) and 6-*epi*-ophiobolin A (**351**) were more phytotoxic than their anhydro derivatives 3-anhydroophiobolin A (**352**) and 3-anhydro-6-*epi*-ophiobolin A (**353**) against sorghum (*Sorghum bicolor*), sicklepod (*Cassia obtusifolia*), and maize (*Zea mays*) [233].

The phytotoxic ophiobolin congeners from fungi were summarized as ophiobolin A (**350**), 6-*epi*-ophiobolin A (**351**), 3-anhydroophiobolin A (**352**), 3-anhydro-6-*epi*-ophiobolin A (**353**), ophiobolin B (**354**), ophiobolin C (**355**), ophiobolin I (**356**), 25-hydroxyophiobolin I (**357**), and ophiobolin J (**358**) [229]. Among them, ophiobolin A (**350**) was proven to be the most phytotoxic to almost all of the tested plants. The structures of 3-anhydroophiobolin A (**352**) and 3-anhydro-6-*epi*-ophiobolin A (**353**) lacked a hydroxyl group at C-3 on the basis of ophiobolin A (**350**) and 6-*epi*-ophiobolin A (**351**), respectively. The hydroxyl (C-3) of ophiobolin A (**350**) improved the inhibition against barley and cabbage, reduced the activity on tested plants, while the activity on the remaining tested plants was unchanged. Furthermore, the stereochemistry at C-6, and the aldehyde group at C-7 were also important for the phytotoxicity of the molecules [234,235]. Ophiobolins have been considered to have the herbicidal potential to control weeds [229,230].

Ophiobolin C (**355**), ophiobolin I (**356**) and 25-hydroxyophiobolin I (**357**) were isolated the cultures of *Drechslera maydis*, the causal agent of Southern corn leaf blight. These compounds were toxic to corn, Johnson grass, and sorghum by leaf-wouding assay [234]. Ophiobolin J (358) was later isolated from *D. maydis*, and showed a similar phytotoxicity [236].

Two bicycle sesterterpenes named terpestacin (**359**) and 11-*epi*-terpestacin (also called siccanol, **360**) were isolated from the cultures of *Bipolaris sorokiniana* NSDR-011. Both metabolites showed inhibition on the root growth of Italian ryerass seedlings [237]. Terpestacin (**359**) was also isolated from *Neufusicoccum batangarum*, the causal agent of the scabby canker of cactus pear (*Opuntia ficus-indica*). Terpestacin (**359**) showed phytotoxicity on either tomato with the leaf puncture assay or cactus pear with cladode puncture assay [74].

### 4.5. Triterpenoids

**Figure 26:**
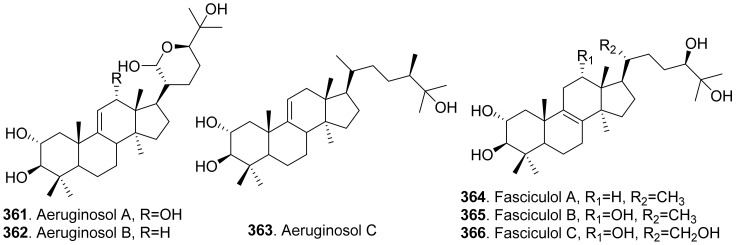
Structures of the phytotoxic triterpenoids isolated from fungi.

Phytotoxic triterpenoids are mainly isolated from the fungi of Basidiomycetes. Their structures are shown in Figure 26. Three lanostane triterpenoids namely aeruginosols A (**361**), B (**362**) and C (**363**) were isolated from the fruiting bodies of *Stropharia aeruginosa*. Among them, aeruginosol C (**362**) showed root growth inhibitory activity on lettuce seedlings [238].

Fasciculols A (**364**), B (**365**) and C (**366**) were isolated from fruiting bodies of *Neamatoloma fasciculare*. They all inhibited root elongation of Chinese cabbage seedlings. The inhibitory activity of fasciculol A (**364**) was only one-fourth of those of fasciculols B (**365**) and C (**366**) [239,240,241].

### 4.6. Meroterpenoids

Meteroterpenoids are natural products that are partially derived from terpenoid biosynthetic pathways. Phytotoxic meteroterpenoids usually contain monoterpene, sesquiterpene, and diterpene biosynthetic pathways.

#### 4.6.1. Meroterpenoids Containing Monoterpene Biosynthetic Pathways

**Figure 27:**
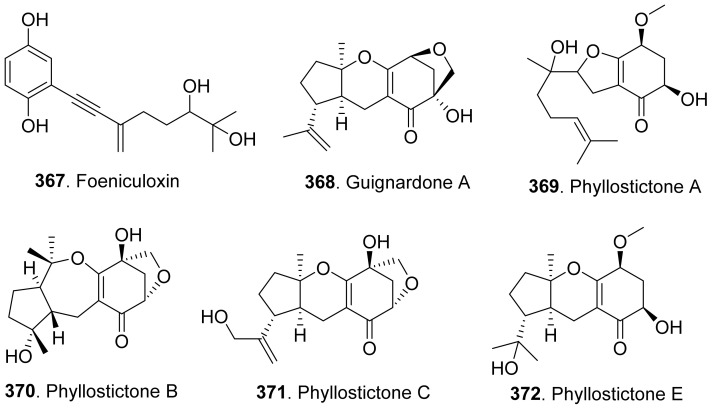

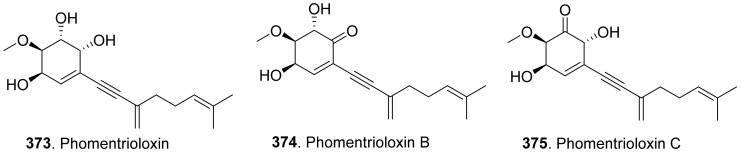
Structures of the phytotoxic meroterpenoids with monoterpene pathways isolated from fungi.

The structues of fungal phytotoxic meroterpenoids contain monoterpene biosynthetic pathways are shown in Figure 27. Foeniculoxin (**367**), a geranylhydroquinone, was isolated from *Phomopsis foeniculi* which was the fungal pathogen (*Phomopsis foeniculi*) of fennel (*Foeniculum vulgare* subsp. *vulgare*) to cause the necrosis of stems, leaves and inflorescences leading to a marked decrease in fruit production [242].

Guignardone A (**368**) was isolated from the culture filtrates of *Macrophomina phaseolina* which was the charcoal rot pathogen of many crops. It was toxic to the non-host plant tomato leaf puncture assay. However, it did not show phytotoxic activity to the host plant soybean [243].

Phyllostictones A-C (**369**-**371**), and E (**372**) were isolated from the endophytic fungus *Phyllosticata capitalensis* derived from the plant *Cephalotaxus fortune*. These three compounds inhibited shoot and root growth of *Lactuca sativa* and *Lolium perenne* seedlings [21].

Phomentrioloxin (**373**), a phytotoxic geranylcyclohexenetriol, was isolated from the liquid culture of *Phomopsis* sp. (teleomorph: *Diaporthe gulyae*) which was isolated from symptomatic saffron thistle (*Carthamus lanatus*). Phomentrioloxin (**373**) causes the appearance of necrotic spots when applied to the leaves of both host and non-host plants. It also caused growth and chlorophyll content reduction of the fronds of *Lemna minor* and inhibition of tomato rootlet elongation [244]. The structure-activity relationship study showed that the hydroxy groups at C-2 and C-4 appeared to be important features for the phytotoxicity, as well as an unchanged cyclohexentriol ring and the unsaturations of the geranyl side chain [245].

Phomentrioloxins B (**374**) and C (**375**) were isolated from *Diaporthe gulyae*, the pathogen of sunflower (*Helianthus annuus*) by causing stem canker. Phomentrioloxin B (**374**) showed small but clear necrotic spots on a number of plant specices when assayed at 5 mM on punctured leaf disks of weedy and crop plants [125].

#### 4.6.2. Meroterpenoids Containing Sesquiterpene Biosynthetic Pathways

**Figure 28:**
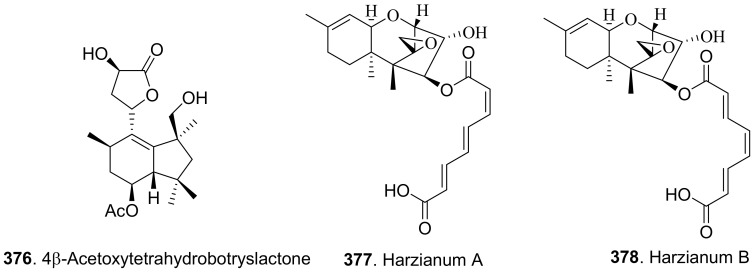

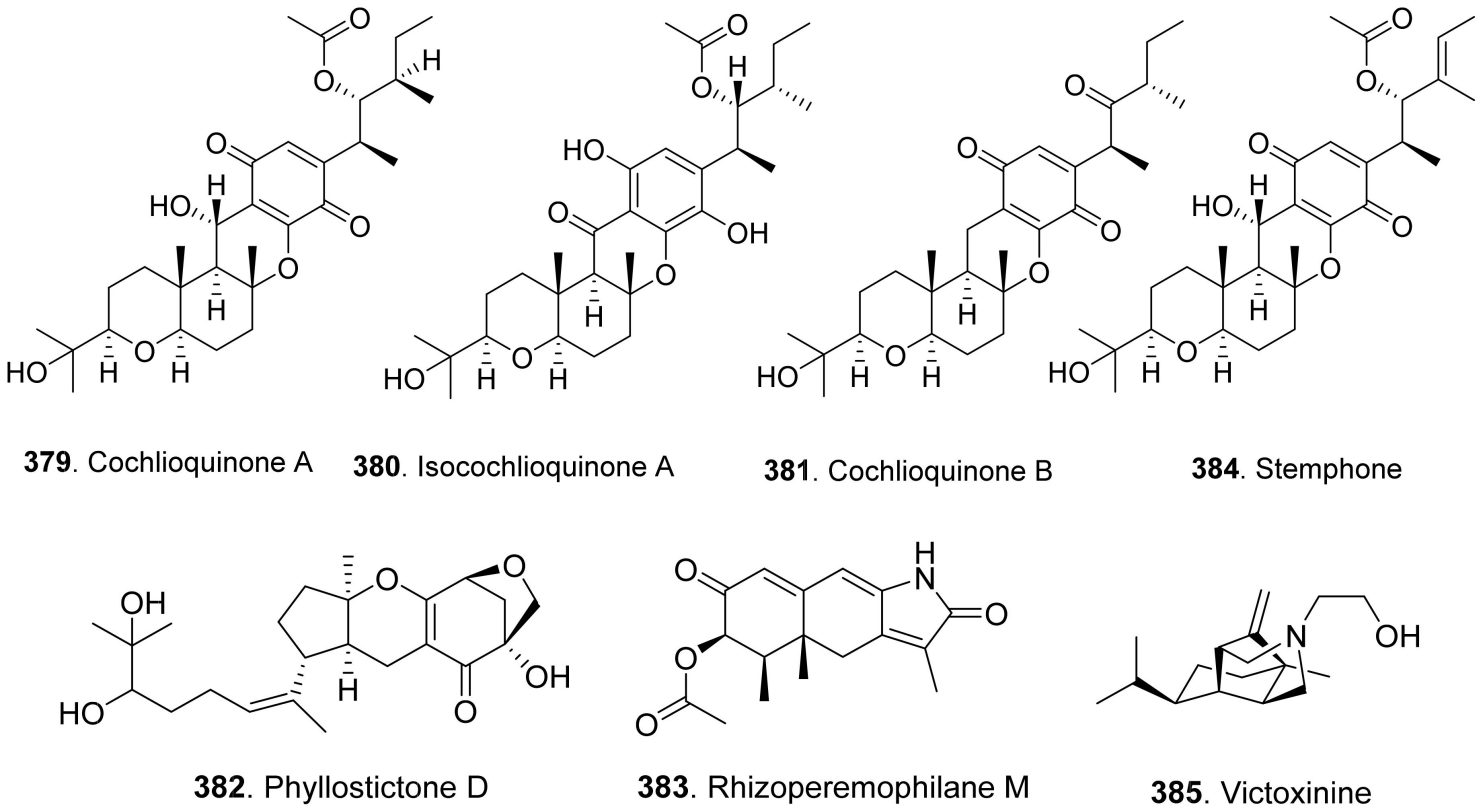
Structures of the phytotoxic meroterpenoids with sesquiterpene pathways isolated from fungi.

The structures of fungal phytotoxic meroterpenoids contain sesquiterpene biosynthetic pathways are shown in Figure 28. 4β-Acetoxytetrahydrobotryslactone (**376**) was isolated from the culture broth of *Botrytis cinerea*. This lactone compound showed a phytotoxic effect on *Phaseolus vulgaris* when tested up to 250 μg/mL by leaf disk assay. It was speculated that the biosynthetic origin of this compound belonged to sesquiterpene-polyketide pathway [246].

Four meroterpenoid quinones cochlioquinons A (**377**) and B (**378**), isocolioquinone A (**379**), and stemphone (**384**) were isolated from the cultures of *Bipolaris bicolor*, the pathogen of gramineous plants such as rice and millet. They inhibited the root growth of the seedlings of finger millet and rice [247]. Their absolute configurations were further elucidated by spectroscopic data interpretation, single-crystal X-ray diffraction analysis, chemical transformations, and biosynthetic considerations [248]. They belonged to polyketide-sesquiterpenoid hybrid compounds biosynthesized through type I polyketide gene cluster by genome sequence analysis of *Bipolaris sorokiniana* [249].

Harzianums A (**380**) and B (**381**) were isolated from the biofertilizer fungus *Trichoderma brevicompactum*. They consisted of the core sesquiterpenoid structure (12,13-epoxytrichothec-9-ene) connected with a linear polyketide-derived substituent (octa-2,4,6-trienedioyl) via an ester bond at C-4. Both harzianums A (**380**) and B (**381**) reduced both shoot and root lengths at low concentrations and inhibited the seed germination of *Brassica chinensis*, *Oryza sativa*, *Echinochloa crusgalli* at 2 μg/mL [250].

Phyllostictone D (**382**) was isolated from the endophytic fungus *Phyllosticata capitalensis* derived from *Cephalotaxus fortune*. This compound inhibited shoot and root growth of *Lactuca sativa* and *Lolium perenne* seedlings [21].

Rhizoperemophilane M (**383**) was a nitrogen-containing eremophilane-type sesquiterpenoids isolated from the endophytic fungus *Rhizopycnis vagum*. This metabolite inhibited radicle elongation of rice seedlings [212].

Victoxinine (**385**) was a nitrogen-containing sesquiterpenoid isolated from *Bipolaris* sp., the pathogen of Johnson grass (*Sorghum halepense*). It was toxic to the leaves of sorghum (*Sorghum bicolor*), sicklepod (*Cassia obtusifolia*), maize (*Zea mays*), morning glory (*Ipomea purpurea*), and bentgrass (*Agrostis alba*) [203].

#### 4.6.3. Meroterpenoids Containing Diterpene Biosynthetic Pathways

**Figure 29:**
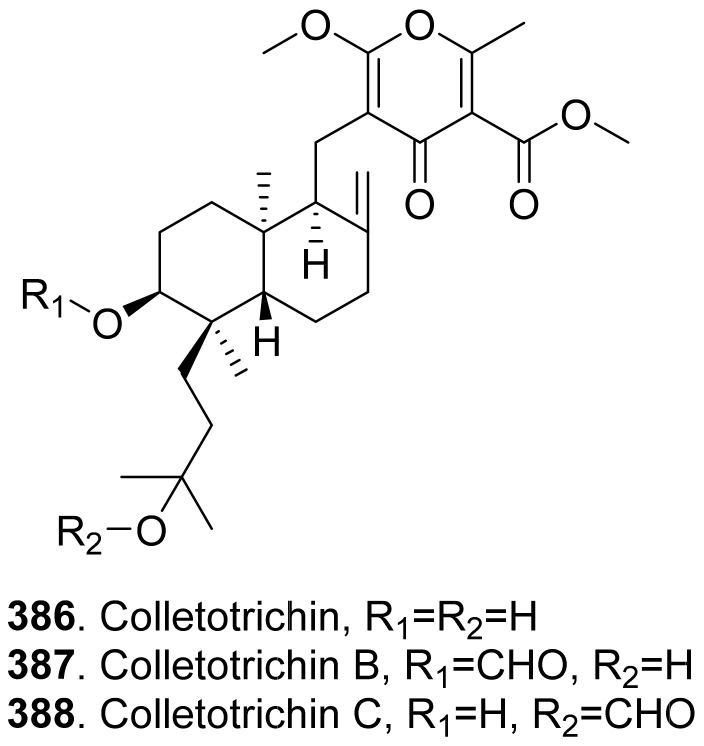
Structures of the phytotoxic meroterpenoids containing diterpene pathways isolated from fungi.

The structures of fungal phytotoxic meroterpenoids contain diterpene biosynthetic pathways are shown in Figure 29. Three meroterpenoids namely colletotrichin (also called colletotrichin A, **386**), colletotrichin B (**387**) and colletotrichin C (**388**) were isolated from the cultures of *Colletotrichum nicotianae*. Their structures all contained a norditerpene and a polysubstituted γ-pyrone. When applied to tobacco leaves, these compounds induced symptoms similar to those of the tobacco anthracnose caused by *C. nicotianae* [121]. They were also toxic to lettuce and rice seedlings [251].

## 5. Nitrogen-Containing Metabolites

Phytotoxic nitrogen-containing metabolites include cyclic peptides, noncyclic oligopeptides, cytochalasins, lactams, indoles, pyridines, amines and noncyclic amides, and others.

### 5.1. Cyclic Peptides

Cyclic peptides are cyclic compounds formed mainly by the amide bonds between either proteinogenic or non-proteinogenic amino acids. Phytotoxic cyclic peptides from fungi mainly include ester bond-containing cyclic peptides (or called cyclic depsipeptides) and ester bond-uncontaining cyclic peptides.

#### 5.1.1. Cyclic Depsipeptides

**Figure 30:**
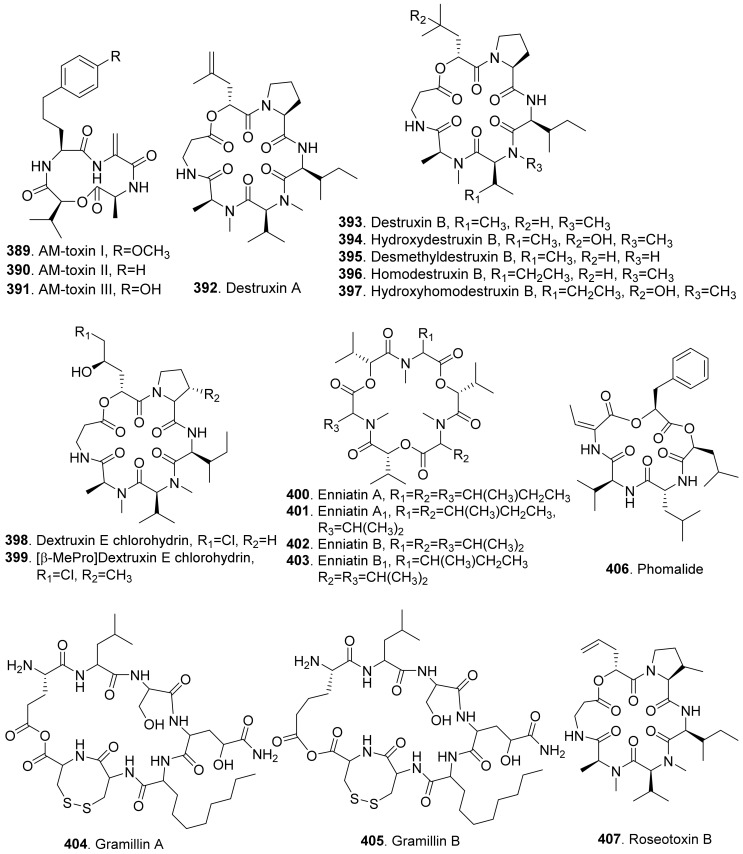
Structures of the phytotoxic cyclic depsipeptides isolated from fungi.

Cyclic depsipeptides (CDPs) are cyclopeptides in which amide groups are replaced by corresponding lactone bonds due to the presence of a hydroxylated carboxylic acid in the peptide structure [252]. The structures of phytotoxic cyclic depsipeptides from fungi are shown in Figure 30.

AM-toxins I (**389**), II (**390**) and III (**391**) belong to cyclic tetradepsipeptides. They were host-specific phytotoxins isolated from *Alternaria mali*, the pathogen of apple blotch disease [253,254]. It was found that AM-toxin I (**389**) inhibited photosynthetic O_2_ evolution in a host-specific manner [255].

Destruxin congeners are cyclic hexadepsipeptides belonging to host-specific phytotoxins. Destruxin A (**392**) was isolated from the culture broth of *Alternaria linicola*, the seed-borne pathogen of linseed (*Linum usitatissimum*). The infected seeds caused poor germination and damping-off of the seedlings. *Alternaria linicola* also caused leaf spotting on seedling and adult plants, and a form of head blight in the seed capsules which resulted in a loss of yield and reduction in oil quality [256]. Three cyclic hexadepsipeptides, namely destruxin B (**393**), desmethyldestruxin B (**395**) and homodestruxin (**396**), were isolated from the culture filtrates of *Alternaria brassicae*, the pathogen responsible for the balck spot of canola. They were assayed on the leaves of host and non-host plants. Dextruxin B (**393**) induced symptoms ranging from severe chlorosis and necrosis to almost no visible chlorosis. Dextruxin B (**393**) was proved as the host specific phytotoxin [257]. Both destruxin B (**393**) and homodestruxin B (**396**) could be transformed to hydroxydestruxin B (**394**) and hydroxyhomodestruxin B (**397**), respectively by host plants. The hydroxylated products (**394** and **397**) were less phytotoxic than their corresponding destruxins. It was considered as the detoxification strategy of canola against *Alternaria* fungi [258].

Two destruxin E derivatives, namely destruxin E chlorohydrin (**398**) and [β-Me-Pro]destruxin E chlorohydrin (**399**) from *Beauveria feline* were screened to have phytotoxic activity against the radicle growth of *Amaranthus retroflexus* seedlings. The structure-activity study showed that chlorine atom played an important role for their phytotoxic activity [28].

Phytotoxic enniatin derivatives included enniatins A (**400**), A1 (**401**), B (**402**), and B1 (**403**). They belong to the class of cyclodepsipeptides found in various *Fusarium* species, and consist of alternating residues of D-2-hydroxyisovaleric acid and a branched chain *N*-methyl L-amino acid, linked by peptide and ester bonds. Enniatins are host non-specific toxins which caused wilt and necrosis during infection of the host, probably related to their ionophoric properties [259]. Enniatins from *Fusarium tricinctum* reduced the growth of germination of wheat seeds [260]. Enniatins might act synergistically as a phytotoxin complex, which caused wilt and necrosis of plant tissue [261]. Enniatin B (**402**) and acetamido-butenolide (**515**) isolated from *Fusarium avenaceum*, the pathogen of spotted knapweed (*Centaurea maculosa*), also acted synergistically to cause necrotic lesions on the leaves of different plant species [262].

Two bicyclic lipopeptides, gramillins A (**404**) and B (**405**), were isolated from *Fusarium graminearum*. They were produced in planta in maize silks by promoting fungal virulence on maize, but had no discernible effect on wheat head infection. Leaf infiltration of the gramillins induced cell death in maize, but not in wheat. This indicated that gramillins were host-specific phytotoxins which were deployed as the virulence agents by *F. graminearum* in maize [263].

Phomalide (**406**) was a host-selective phytotoxin isolated from the virulent isolates of *Leptoshphaeria maclans*. It was a cyclic pentadepsipetide with three α-mino acids and two α-hydroxy acids. Phomalide (**406**) caused disease symptoms (necrotic, chlorotic, and reddish lesions) on canola, but not on either brown or white mustard [264].

Roseotoxin B (**407**) was isolated from *Trichothecium roseum*, the pathogen of apple pathogen. This metabolite was able to penetrate apple peel and produced chlorotic lesions by using kinetic fluorescence imaging method. It was the direct evidence of phytopathogenic activity of reseotoxin B (**407**) of *Trichothecium roseum* on apple [265].

#### 5.1.2. Cyclic Peptides without Ester Bond

**Figure 31:**
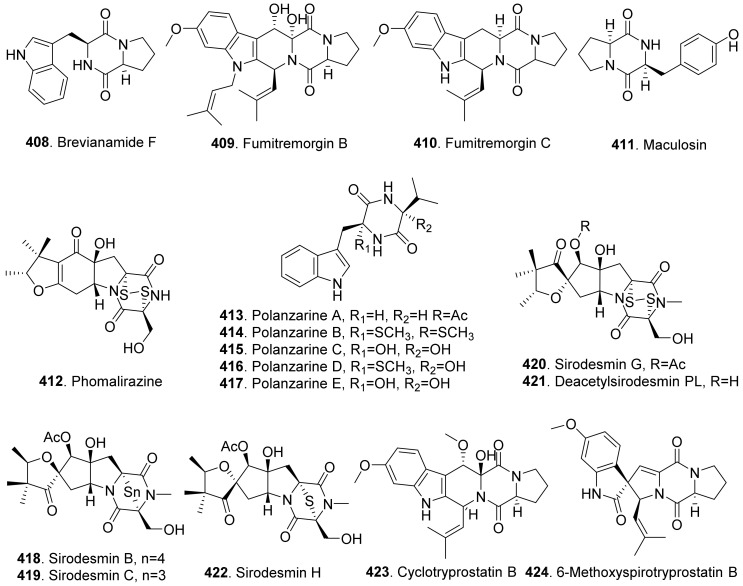

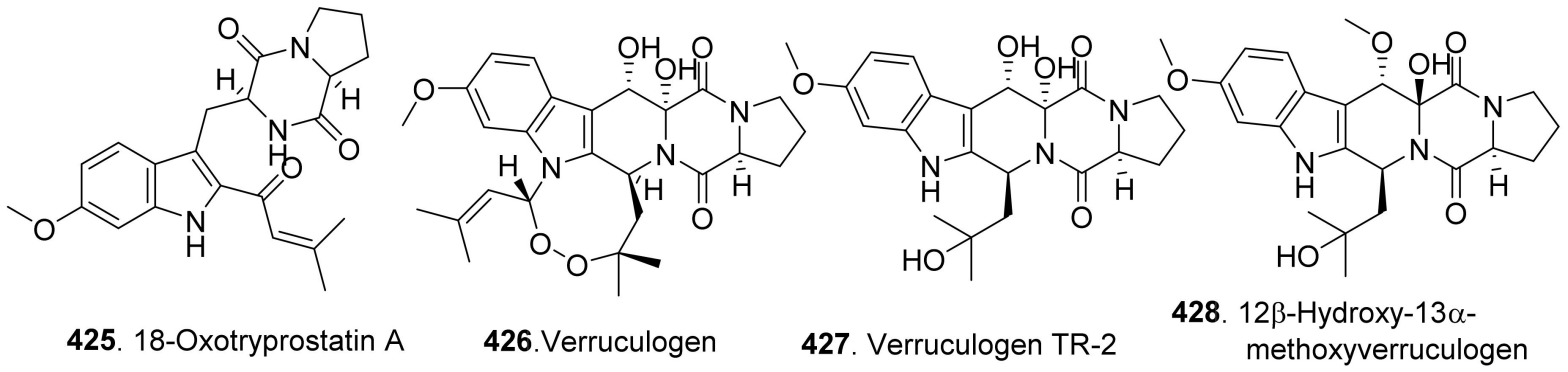
Structures of the phytotoxic cyclodipeptides isolated from fungi.

Cyclic peptides without ester bond are cyclic metabolites formed only by proteinogenic or non-protenogenic amino acids joined together by amide bonds (or peptide bonds). They mainly include cyclic di-, tri-, tetra-, penta-, hexa-, hepta-, octa-, nona-, and decapeptides [266,267]. Among them, cyclodipeptides are the most important group. Cyclodipeptides are also called 2,5-diketopiperazines or dioxopiperazines [266]. The structures of phytotoxic cyclodipeptides from fungi are shown in Figure 31.

Brevianamide F (also called cyclo(_L_-Trp-_L_-Pro), **408**), fumitremorgin B (**409**), fumitremorgin C (**410**), cyclotryprostatin B (**423**), 6-methoxyspirotryprostatin B (**424**), 18-oxotryprostatin A (**425**), verruculogen (**426**), verruculogen TR-2 (also called TR-2, **427**), and 12β-hydroxy-13α-methoxyverruculogen TR-2 (**428**) were tryptophan-proline cyclodipeptides isolated the endophytic fungus *Aspergillus fumigatus* derived from the plant *Melia azedarach*. They inhibited shoot and root elongation of the seedlings of turnip (*Raphanus sativus*) and amaranth (*Amaranthus mangostanus*) [268].

Maculosin (**411**) was a host-specific phytotoxin isolated from *Alternaria alternata*, the pathogen of black leaf blight disease of spotted knapweed (*Centaurea maculosa*) [93,269].

Phomalirazine (**412**) was isolated from *Leptosphaeria maculans*, the pathogen of canola. This compound was to toxic to canola and brown mustard by leaf puncture assay [270].

Polanrazines A (**413**), B (**414**), C (**415**), D (**416**) and E (**417**) were isolated from Polish isolates of *Leptosphaeria maculans*, and showed moderate toxicity on the leaves of brown mustard [271].

Phytotoxic sirodesmin congeners such as sirodesmins B (**418**), C (**419**), G (also called sirodesmin PL, **420**), and H (**422**), and deacetylsirodesmin PL (**421**) were isolated from cultures of *Leptosphaeria maculans*, the blackleg canker pathogen of oilseed rape (*Brassica napus*). The phytotoxicity was partly due to the sulfur bridge [270,272,273].

**Figure 32:**
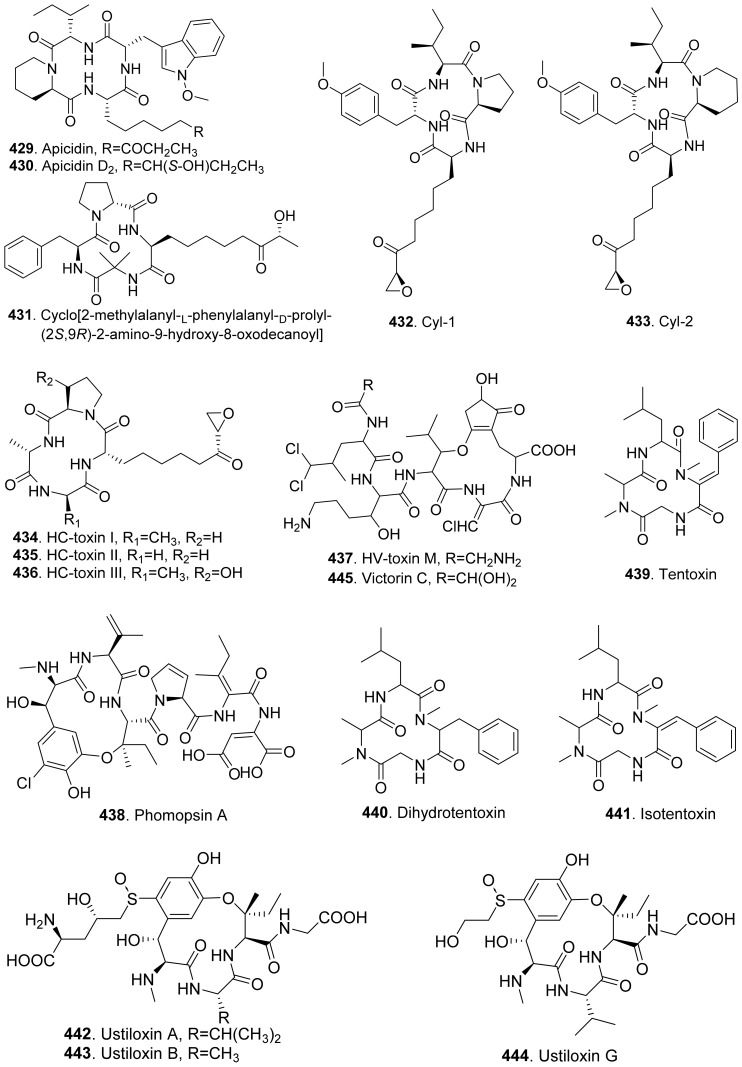
Structures of the phytotoxic and ester bond-free cyclic peptides, excluding cyclodipeptides isolated from fungi.

The structures of phytotoxic and ester bond-free cyclic peptides, excluding cyclodipeptides from fungi, are shown in Figure 32. Two cyclic tetrapeptides named apicidin (**429**) and apicidin D_2_ (**430**) were isolated from *Fusarium semitectum* KCTC16676, which was isolated from soybean seeds. Both apicidin (**429**) and apicidin D_2_ (**430**) showed inhibition on seedling growth of six plant species including maize, cucumber, lettuce, soybean, tomato, and wheat [274].

Cyclo[2-methylalanyl- _L_-phenylalanyl-_D_-prolyl- (2*S*,9*R*)-2-amino-9-hydroxy- 8-oxodecanoyl] (**431**), a phytotoxic cyclotetrapeptide, was isolated from the culture broth of *Verticillium coccosporum*. This metabolite was toxic to *Lemna minor* [275].

Cyl-1 (**432**) and Cyl-2 (**433**) from the culture broth of *Cylindrocladium scoparium*, the pathogen of many higher plant diseases, inhibited root growth of lettuce seedlings [276]. Phytotoxic Cyl-1 (**432**) and Cyl-2 (**433**) were previously isolated from *C. scoparium*, respevtively [277,278]

HC-toxin I (also called HC-toxin, **434**) was a host-specific phytotoxin produced by *Helminthosporium carbonum* (syn. *Cochliobolus carbonum*), the pathogen to cause necrotic lesions on maize leaves [279]. HC-toxins II (**435**) and III (**436**) along with HC-toxin I (**434**) were latter isolated from *H. victoriae*. These three HV-toxins were considered as the host-specific toxins to show root growth inhibition on the susceptible maize seedlings [280].

HV-toxin M (**437**) was another host specific phytotoxin isolated from the culture broth of *Helminthosporium victoriae*, the causal agent of victoria blight disease of oat [281].

Phomopsin A (**438**) was a cyclic tripeptide with a tripeptide side chain isolated from *Phomopsis leptostromiformis*. This compound inhibited seedling growth of lupinis [282,283].

Tentoxin (**439**) and dihydrotentoxin (**440**) were isolated from the culture filtrates of *Alternaria alternata*. Both metabolites caused distinct wilting of the cuttings of weed *Galium aparine* [284,285,286]. Tentoxin (**439**) was also isolated from the cultures of *Alternaria linicola*, the seed-borne pathogen of linseed (*Linum usitatissimum*) by causing poor germination and damping-off of the seedlings [256]. Isotentoxin (**441**), the *E*-isomer of tentoxin (**439**), was produced from tentoxin (**439**) by UV-irradiation. The transformed isotentoxin had stronger wilting effects against the weed *Galium aparine* than tentoxin (**439**) [285,286].

Ustiloxins A (**442**), B (**443**) and G (**444**) were isolated from *Ustilaginoidea virens* (teleomorph: *Villosiclava virens*), the pathogen of rice false smut disease. They showed strong inhibition on the radicle and germ elongation of rice seedlings. When their concentrations were at 200 μg/mL, the inhibitory ratios of radicle and germ elongation were more than 90% and 50%, respectively, the same effect as that of positive control (glyphosate). They also induced abnormal swelling of the roots and germs of rice seedlings [267].

Victorin C (**445**) was a host-specific phytotoxin from *Cochliobolus victoriae* (syn. *Helminthosporium victoria*), the causal agent of victoria blight of oats [287].

### 5.2. Noncyclic Oligopeptides

**Figure 33:**
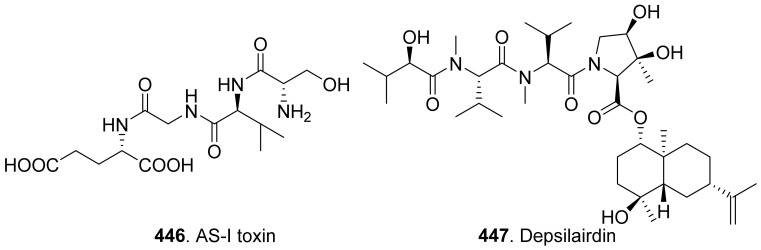
Structures of the phytotoxic noncyclic oligopeptides isolated from fungi.

Fungal phytotoxic noncyclic oligopeptides are linear compounds composed of several amino acids (Figure 33). AS-I toxin (**446**) was a phytotoxic tetrapeptide (Ser-Val-Gly-Glu) isolated from the culture filtrates of *Alternaria alternata*, the pathogen of sunflower (*Helianthus annuus*) by causing leaf necrotic spots. AS-I toxin (**446**) was toxic to sunflower. Nontoxic or very slight toxic effects were observed on the other tested plants which indicated that AS-I toxin (**446**) was a host-specific phytotoxin [288].

Depsilairdin (**447**) produced by *Leptosphaeria maculans* possessed a tripeptide coupled with a sesquiterpene moiety. Depsilairdin (**447**) caused disease symptoms similar to those caused by the pathogen. Plant leaves of brown mustard treated with depsilairdin (**447**) showed strong necrotic and chlorotic lesions, but such symptoms were not observed in canola at a wide concentration range from 1 μM to 1 mM [289].

### 5.3. Cytochalasin Congeners

**Figure 34:**
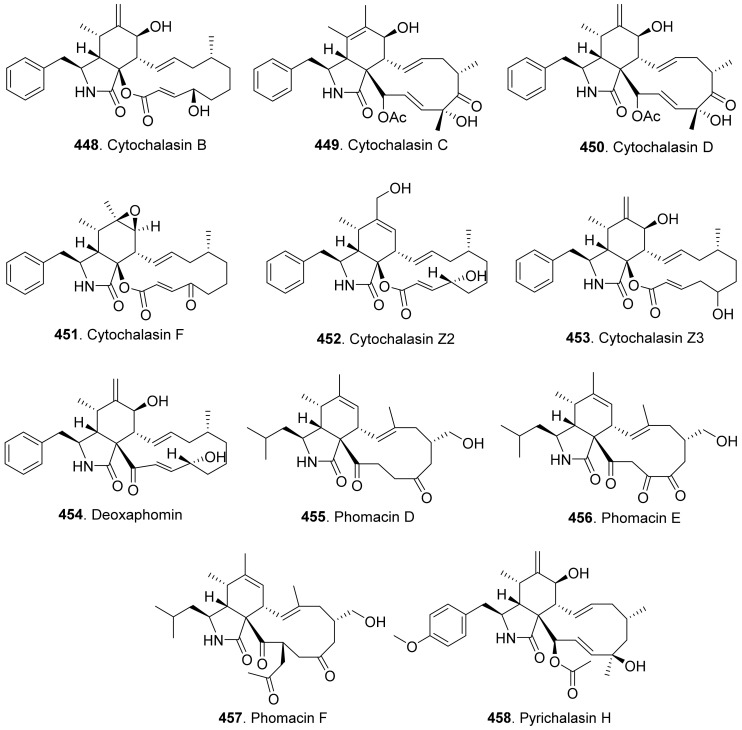
Structures of the phytotoxic cytochalasins isolated from fungi.

Cytochalasins belong to class of perhydroisoindolylamcrocylclic lactones. The structures of phytotoxic cytochalasin congeners from fungi are shown in Figure 34. Cytochalasin B (**448**) was isolated from the culture filtrates of the plant pathogens *Drechslera wirrenganensis* and *D. campanulata*, and was toxic to the leaves of faba bean by leaf puncture assay [290].

Cytochalasins C (**449**) and D (**450**) were isolated from the endophytic fungus *Xylaria cubensis* associated with *Eugenia brasiliensis* (Myrtaceae). Both cytochalasins showed phytotoxic activity on wheat coleoptiles [291]. Cytochalasin D (**450**) was also isolated from the culture filtrates of *Ascochyta rabiei* (teleomorph: *Didymella rabiei*), the pathogen of chickpea. This metabolite was toxic to the leaflet cells of chickpea [292].

Five cytochalasin analogues named cytochalasins B (**448**), F (**451**), Z2 (**452**), Z3 (**453**) and deoxaphomin (**454**) were isolated from the *Phoma exigua* var. *exigua* (syn. *Ascohyta sonchi*), the leaf pathogen of *Cirlum arvense* and *Sonchus arvensis*. These cytochalasin analogues were all toxic to the leaves of *Cirlum arvense* and *Sonchus arvensis* by leaf disk-puncture assay [293].

Three cytochalasins named phomacins D (**455**), E (**456**) and F (**457**) were identified from the wheat pathogen *Parastagoospora nodorum* by genomics-driven discovery. Both phomacins D (**455**) and E (**456**) obviously inhibited wheat seed germination at 100 μg/mL. Phomacin F (**457**) just had week inhibitory activity on wheat seed germination. Interestingly, phomacin D (**455**) did not show any inhibition of seed germination against the dicots *Arabidopsis thaliana* and *Lepidium sativum*, which indicated that seed germination inhibition of phomacin D (**455**) could be specific to monocots [294].

Pyrichalasin H (**458**) was isolated from the cultures of *Pyricularia grisea*, the causative fungus of blast disease in crabgrass (*Digitaria sanguinalis*). This compound strongly inhibited growth of rice seedlings at 1 μg/mL [295].

### 5.4. Lactams

**Figure 35:**
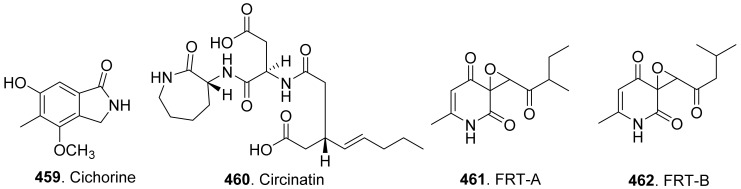

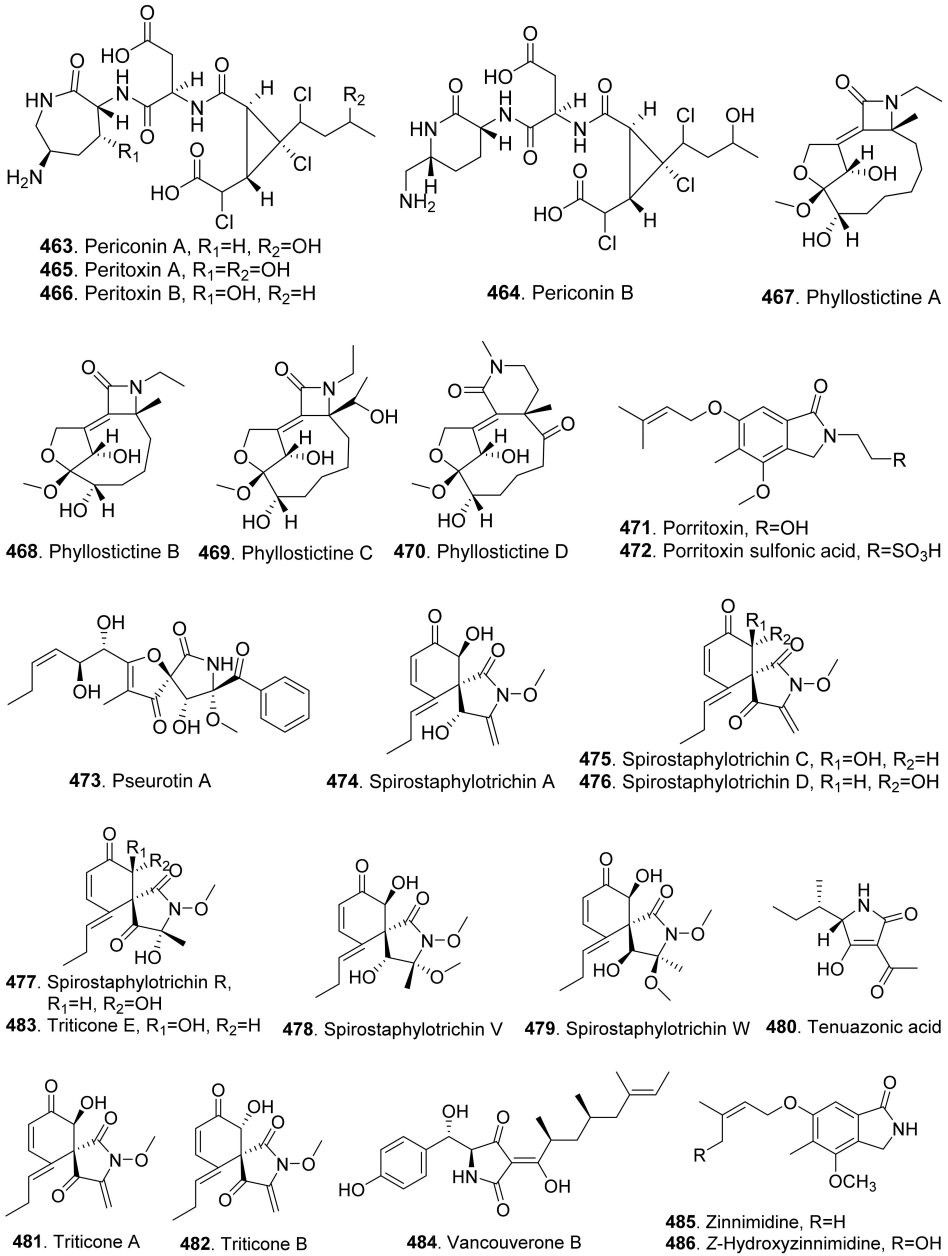
Structures of the phytotoxic lactams isolated from fungi.

The structures of phytotoxic lactams from fungi are shown in Figure 35. Cichorine (**459**), zinnimidine (**485**) and *Z*-hydroxyzinnimidine (**486**) were isolated from the fungus *Alternaria cichorii*, the pathogen of foliar blight disease of Russian knapweed (*Acroptilon repens*). These compounds were toxic to the leaves of Russian knapweed by in vitro leaf puncture assay [185].

Two apple fruit rot toxins namely FRT-A (also called sapinopyridione, **461**) and FRT-B (also called flavipucine, **462**) were isolated from *Botryosphaeria berengeriana* (anamorph: *Macrophoma* sp.). Both compounds were isomers and showed necrosis-inducing activity on apple fruits by dropping assay [296]. FRT-A (**461**) was also found in *Sphaeropsis sapinea*, the fungal pathogen of conifers occurring world-wide. It was toxic to three cypress species (*Cupressus arizonica*, *C. macrocarpa*, and *C. sempervrens*) by cutting twig assay [297]. The 3:1 mixture of FRT-A (**461**) and FRT-B (**462**) showed strong phytotoxic activity to plants bentgrass (*Agrostis stolonifera*) and lettuce (*Lactuca sativa*) by seedling growth assay [298].

Five metabolites named circinatin (**460**), periconins A (**463**) and B (**464**), and peritoxins A (**465**) and B (**466**) were isolated from the culture broth of *Periconia circinata*. Peritoxins A (**465**) and B (**466**) inhibited root growth of the susceptible genotype of sorghum by 50% at 1–4 ng/mL. Periconin A (**463**) showed weak inhibition on sorghum root growth, and no activity could be detected for circinatin (**460**) and periconin B (**464**) [299].

Four oxazatricycloalkenones phyllostictines A (**467**), B (**468**), C (**469**) and D (**470**) isolated from *Phyllosticta cirsii* showed phytotoxic to *Cirsium arvense*. Phyllostictine A (**467**) was proved to be highly toxic. Phyllostictines B (**468**) and D (**470**) were slightly less toxic compared to phlyllostictine A (**467**), wheras phyllostictine C (**469**) was almost not toxic, that showed a clear structure-activity relationship between the phytotoxic activity and the structural features characterizing phyllostictine group. Phyllostictine A (**467**) should be potential mycoherbicide for *Cirsium arvense* biocontrol [300].

Porritoxin (**471**) was first identified as a benzoxazocine derivative from the culture broth of *Alternaria porri*, the causal pathogen of black spot disease in stone-leek and onion [301]. The structure of porritoxin (**471**) was then corrected as isoindol-1-one congener [302]. This compound inhibited shoot and root growth of lettuce seedlings at 10 μg/mL [301]. Another isoindo-1-one, namely porritoxin sulfonic acid (**472**) was later isolated from *A. porri*. Structure-phytotoxicity investigation showed that the *N*-alkyl and hydroxyl groups contributed to the phytotocitiy, but that this activity became weak with sulfonation [286].

Pseurotin A (**473**) was produced by *Ascochyta lentis*. This metabolite was phytotoxic to lentil (*Lens culinaris*), and was light-dependent [87].

Spirostaphylotrichins A (**474**), C (**475**), D (**476**), R (**477**), V (**478**) and W (**479**) as well as triticone E (**483**) were isolated from *Pyrenophora semeniperda* (anamorph: *Drechelera* sp.), the seed pathogen of cheatgrass (*Bromus tectorum*). This compound was toxic to the leaves of wheat, tomato, sowthistle and cheatgrass [303].

Tenuazonic acid (also called TeA, TA, and AAC-toxin, **480**) was a tetramic acid phytotoxin which was isolated from a series of *Alternaira* fungi such as *A. alternata* [304], *A. citri* [305], *A. crassa* [306], *A. linicola* [256], *A. tenuissima* [307]. This compound was also produced by other plant pathogenic fungi such as *Magnaporthe oryzae* [308] and *Phoma sorghina* [309]. Tenuazonic acid (**480**) was found to inhibit photosynthesis with the target site as photosystem II (PSII) [310,311,312]. Therefore, tenuazonic acid (**480**) was considered as the potential herbicide [304,313].

Triticones A (**481**) and B (**482**) were two spirocyclic γ-lacams isolated from *Drechslera tritici-repentis*, the causal agent of reddish brown spots on wheat (*Triticum vulgare*). Two compounds in mixture showed phytotoxicity on the leaves and protoplasts of wheat [314].

Vancouverone B (**484**) was isolated from a sea snail-derived fungus, *Penicillium vancouverense* YY-1. This metabolite exhibited inhibition on the seedling root growth of *Lactuca sativa*, *Trifolium repens*, *Rahanus sativus* var. *longipinnatus*, and *Brassica rapa* var. *perviridis* [315].

### 5.5. Indole Derivatives

**Figure 36:**
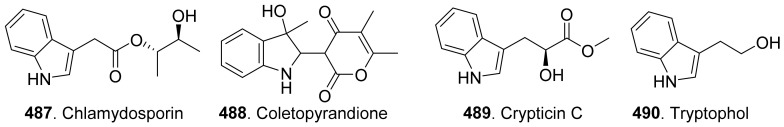
Structures of the phytotoxic indole derivatives isolated from fungi.

The structures of phytotoxic indole derivatives from fungi are shown in Figure 36. Chlamydosporin (**487**) was isolated from the endophytic fungus *Fusarium chlamydosporum* residing in the roots of *Suaeda glauca*. This indole derivative exhibited significant phytotoxic activity against the radicle growth of *Echinochloa crusgalli* seedlings with the inhibition rate of more than 80%, even at concentration of 1.25 μg/mL [316].

Colletophyrandione (**488**) was a tetrasubstituted indolyllidenepyrandioine isolated from the culture filtrates of *Colletotrichum higginsianum*. It was toxic to four plant species *Sonchus arvensis*, *Helianthus annuus*, *Convolulus arvensis*, *Ambrosia artemisiifolia* by leaf puncture assay [317].

Crypticin C (**489**) was isolated from the culture filtrates of *Diaporthella cryptica*, the emerging hazelnut pathogen. This compound was active in the tomato cutting assay [173].

Tryptophol (**490**) was isolated as a major metabolite from the cultures of *Drechslera nodulosum* (syn. *Helminthosporium nodulosum*), the pathogen of seedling blight of goosegrass (*Eleusine indica*). This metabolite caused necrotic lesions on goosegrass at 6.2 × 10^−4^ M [318].

### 5.6. Pyridine Derivatives

**Figure 37:**
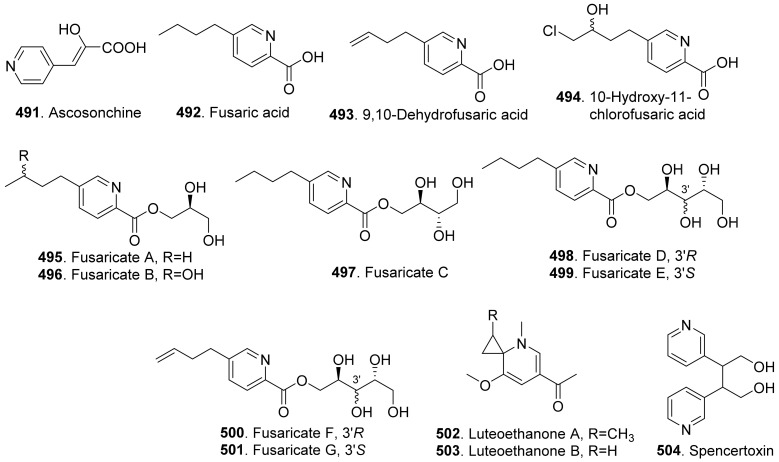
Structures of the phytotoxic pyridine derivatives isolated from fungi.

The structures of phytotoxic pyridine derivatives from fungi are shown in Figure 37. Ascosonchine (**491**) was the enol tautomer of 4-pyridylpyruvic acid with herbicidal activity produced by *Ascohyta sonchi,* the leaf pathogen of *Sonchus arvensis*, a perennial herbaceous weed occurring throughout the temperate regions of the world. Ascosonchine (**491**) was toxic to *Sonchus arvensis* and showed selective herbicidal properties [319].

Fusaric acid (also called 5-butylpicolinic acid, **492**) was isolated from *Fusarium oxysporum*. It was toxic to tobacco leaves by pucture assasy [320]. Fusaric acid (**492**) was produced by several *Fusarium* species which commonly infected cereal grains and other agricultural commodities [321]. Both fusaric aicd (**492**) and 9,10-dehydrofusaric acid (**493**) were isolated from *Fusarium nygamai*, which caused large leaf and stem necrosis on the host *Striga hermonthica*. These two compounds showed a wide chlorosis and necrosis in the punctured aera of tomato leaves as well as a strong inhibition on root elongation of tomato seedlings [322].

The endophytic fungus *Fusarium oxysporum* from the fruits of *Drepanocarpus lunatus* affored eight new fusaric acid derivatives, 10-hydroxy-11-chlorofusaric acid (**494**), and fusaricates A–G (**495**-**501**). All isolated fusaric acid derivatives showed significant phytotoxicity to the leaves of barley [323].

Luteoethanones A (**502**) and B (**503**), two 1-substituted ethanones, were isolated from *Neofusicoccum luteum*, the causal agent of Botryosphaeria dieback on grapevine. Both metabolites caused large necrotic spots, severe shriveling, and distortion of the leaf lamina of grapevine by leaf detached assay [39].

Spencertoxin (**504**), a dipyridine-butane-1,4-diol derivative, was isolated from *Spencermartinsia viticola*, the causal agent of Botryosphaeria dieback on grapevine in Australia. Spencertoxin (**504**) showed phytotoxicity on *Vitis lambrausca* and *V. vinifera* cv. Shiraz by grapevine leaf assay [163].

### 5.7. Amines and Noncyclic Amides

**Figure 38:**
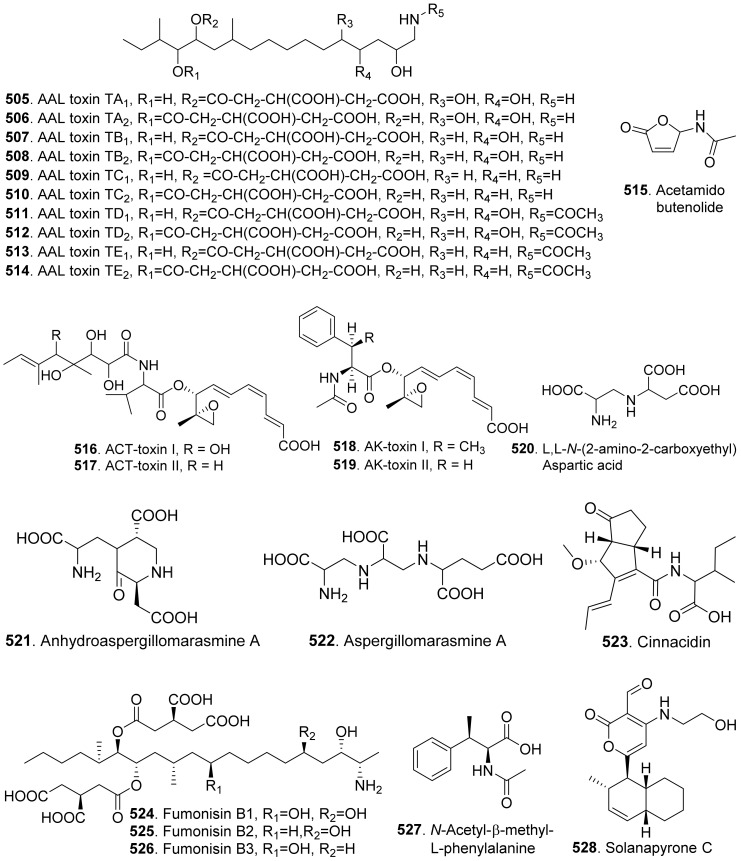
Structures of the phytotoxic amines and noncyclic amides isolated from fungi.

Phytotoxic amines and noncyclic amides are the common nitrogen-containing metabolites produced by fungi. Their structures are shown in Figure 38. Ten AAL toxins, TA_1_ (**505**), TA_2_ (**506**), TB_1_ (**507**), TB_2_ (**508**), TC_1_ (**509**), TC_2_ (**510**), TD_1_ (**511**), TD_2_ (**512**), TE_1_ (**513**), and TE_2_ (**514**), were isolated from *Alternaria alternata* f.sp. *lycopersici*, the pathogen of tomato stem canker disease [324,325,326]. These metabolites consisted of tricarballylic acid esters at either C_13_ or C_14_ of an amino polyol backbone. They were toxic to all tissures of tomato cultivars at low concentrations and induced apoptosis in sensitive tomato plants [327]. They were considered as the host-specific toxins (HSTs) as they were toxic to host plants and had lower phytotoxicity to non-host plants. Further action mechanism study showed these AAL toxins inhibited *de novo* sphingolipid (or called ceramide) biosynthesis of tomato. Therefore, AAL toxins were called sphinganine-analog mycotoxins (SAMs) [328].

Acetamido-butenolide (**515**) was isolated from *Fusarium avenaceum*, the pathogen of spotted knapweed (*Centaurea maculosa*), acted synergistically with enniatin B (**402**) to cause necrotic lesions on leaves of different plant species [262].

ACT-toxins I (**516**) and II (**517**) were isolated from *Alternaria citri* (previously as *A. alternata*), the pathogen of tangerines and mandarins (*Citrus reticulata*). They were also host-specific toxins [329].

AK-toxins I (**518**) and II (**519**), another two host-specific toxins, were isolated from *Alternaria kikuchiana* (previously as *A. alternata*), the pathogen of either apple leaf spot disease or Japanese pear black spot disease [330].

L,L-*N*-(2-amino-2-carboxyethyl) aspartic acid (**520**), anhydroaspergillomarasmine A (**521**) and aspergillomarasmine A (**522**) were isolated as phytotoxic metabolites from the liquid culture of *Pyrenophora teres* (anamorph: *Drechslera teres*, syn: *Helminthosporium teres*), the causal agent of net bloch of barley, a serious disease common in humid and temperate climates. Tested on detached barley leaves, L,L-*N*-(2-amino-2-carboxyethyl) aspartic acid (**520**), and aspergillomarasmine A (**522**) induced chlorotic and necrotic symptoms, while only aspergillomarasmine A (**522**) showed weak phytotoxicity [331].

Cinnacidin (**523**), a cyclopentalenne-isoleucine, was isolated from *Nectria* sp. This metabolite inhibited the root growth of *Arabidopis thaliana* and bentgrass seedlings [332].

Fumonisins B1 (FB1, **524**), B2 (FB2, **525**), and B3 (FB3, **526**) were isolated from the cultures of *Fusarium moniliforme*. They were long-chain polyhydroxyl alkylamines with two propane tricarboxylic acid moieties esterified to hydroxyls on adjacent carbons. These compounds inhibited seedling growth of corn and tomato [333].

*N*-Acetyl-β-methyl-L-phenylalanine (**527**) was isolated from *Villosiclava virens* (anamorph: *Ustilaginoidea virens*), the pathogen of rice false smut disease. It inhibited rice radicle elongation [334].

Solanapyrone C (**528**) has been isolated from the culture filtrate of *Alternaria solani*, the causal organism of early blight disease of tomato and potato [132], and the culture filtrates of *Ascochyta rabiei*, the pathogen of chickpea [133]. Solanapyrone C (**528**) was toxic to the leaves of the host plants.

### 5.8. Other Nitrogen-Containing Metabolites

**Figure 39:**
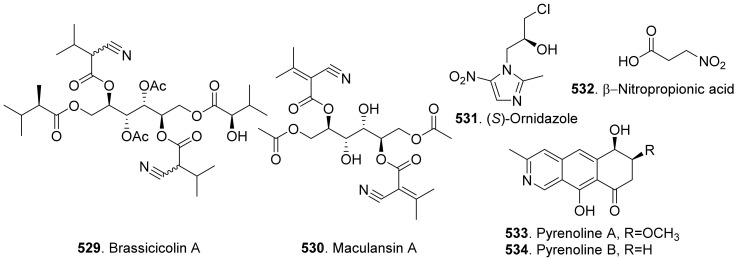
Structures of the other phytotoxic nitrogen-containing metabolites isolated from fungi.

The structures of other fungal phytotoxic nitrogen-containing metabolites are shown in Figure 39. Brasicicolin A (**529**) was isolated from *Alternaria brassicola*, the dark leaf spot pathogen of *Brassica* species. Brasicicolin A (**529**) was a polyester of mannitol esterified with two α-isocyanoisopentanoyl, two α-hydroxyisopentanoyl and two acetyl residues. It was a mixture of diastereomers due to the epimerizable protons adjacent to the isocyano group. Brasicicolin A (**529**) was host specific phytotoxin by causing chlorosis and necrosis on the leaves of brown mustard (*Brassica juncea* cv. Cutlass, susceptible) at 0.5 mM, but no detectable damage on the leaves of white mustard (*Sinapis alba* cv. Ochre, resistant) [335].

Maculansin A (**530**) was isolated from *Leptosphaeria maculans* (anamorph: *Phoma lingam*) cultured in potato dextrose broth (PDB) at high temperature. The structure of maculansin A (**530**) was similar to that brasicicolin A (**529**). Maculansin A (**530**) was more toxic to resistant plant (brown mustard) than to susceptible plant (canola) [167].

(*S*)-Ornidazole (**531**), a nitroimidazole, was isolated from the solid culture of *Penicillium purpurogenum* derived from soil. This compound inhibited root and hypocotyl growth of radish seedlings at 100 μM [26].

β-Nitropropionic acid (**532**) was isolated from *Septoria cirsii*, the pathogen of weed Canada thistle (*Cirsium arvense*) growing in virturally all temperate areas of the world. This compound inhibited seed germination and root elongation, and caused the typical symptoms of chlorosis and necrosis on the leaves of Canada thistle and other test plants [336].

Two isoquinoline derivatives named pyrenolines A (**533**) and B (**534**) were isolated from the cultures of *Pyrenophora teres*, the pathogen of barley. Both compounds were toxic to both monocots and dicots by leaf puncture assay [337].

## 6. Miscellaneous

**Figure 40:**
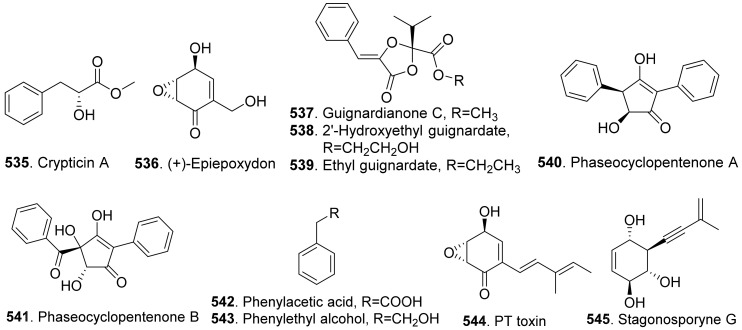
Structures of the miscellaneous phytotoxic metabolites isolated from fungi.

The structures of miscellaneous phytotoxic metabolites from fungi are shown in Figure 40. Crypticin A (**535**), a phenylpropanoid, was isolated from the culture filtrates of *Diaporthella cryptica*, the pathogen of hazelnut. This compound was also called 2-hydroxy-3-phenylpropanoate methyl ester. It was found to be inactive at 1 mg/mL on the leaves of cork oak, grapevine, hazelnut, and holm oak by leaf puncture assay [173].

Two cyclohexene epoxides named (+)-epiepoxydon (**536**) and PT-toxin (**544**) were isolated from *Pestalotiopsis longiseta* and *P. theae*. They induced leaf necrosis on the test leaves [338].

Guignardianone C (**537**), 2′-hydroxyethyl guignardate (**538**) and ethyl guignardate (**539**) were isolated from the endophytic fungus *Phyllosticata capitalensis* derived from *Cephalotaxus fortune*. These three compounds inhibited shoot and root growth of *Lactuca sativa* and *Lolium perenne* seedlings [21].

Phaseocyclopentenones A (**540**) and B (**541**) are penta- and tetrasubstituted cyclopentenones isolated from the culture filtrates of *Macrophomina phaseolina*, the charcoal rot pathogen of many crops. Both compounds were toxic to the non-host plant tomato (*Solanum lycopersicum)* by leaf puncture assay and seedling cutting assay. However, they did not show phytotoxic activity to the host plant soybean (*Glycine max*) [243].

Phenylacetic acid (**542**) was isolated from the liquid culture filtrates by *Biscogniauxia mediterranea*, the pathogen of cork oak (*Quercus suber*). This compound caused epinasty on cork oak cuttings, and wilting on non-host tomato [137].

Phenylethyl alcohol (**543**) was identified in the volatile organic compounds from the endophytic fungus *Hypoxy anthochroum* strain Blaci isolated from *Bursera lancifolia* (Burseaceae). This compound showed phytotoxic activity on seed germination, root elongation and seedling respiration of *Amaranthus hypochondriacus*, *Panicum miliaceum*, *Trifolium pratense* and *Medicago sativa* [186].

Stagonosporyne G (**545**) was an oxygenated acetylenic cyclohexanoid isolated from the *Parastagonospora nodorum* SN15, the pathogen of wheat. This compound displayed a significant phytotoxic activity by killing *Arabidopsis thaliana* seedlings [339].

## 7. Conclusions and Future Perspectives

Due to long-term co-evolution of pathogenic fungi and their host plants, the fungus has evolved strategies for successful infection of host. Among these strategies is the production of phytotoxins. Fungal phytotoxins play an important role in the process of pathogenesis as the mediators of virulenece [12]. They are either host specific or non-host specific. This is why many phytotoxins have been identified from phytopathogenic fungi. In fact, fungal phytotoxins play diverse roles in plant disease, from impacting symptom expression, disease progression, to being required for pathogenesis. Some phytotoxins are directly toxic by killing plant cells and allowing for infection of dead cells. Others interfere with the induction of defense responses, or induce programmed cell death-mediated defense responses in order to generate necrosis required for pathogenicity [7,167,335].

In recent years, more and more phytotoxic metabolites have been discovered from other fungi such as plant and animal endophytic fungi, soil-derived fungi, and marine-derived fungi [21,22,23,24,27,28]. Most of these phytotoxic metabolites are non-host specific. They are suitable for development of herbicides with a broad spectrum. In addition, the phytotoxins from weed pathogens are a very promising source of specific herbicide development for weed control [25,340,341]. An example of success is the discovery of ophiobolins which have shown their potential as herbicides [229,230].

Some isolated fungal phytotoxins can be used as the lead compounds, allowing more toxic compounds based on their structures to be chemically synthesized. An example of this is the synthesis of cinnacidin (**523**) analogs. Two new structural analogs of cinnacidin (**523**), namely (2*S*,3*S*)-2- [(3*RS*,3a*SR*,6a*RS*) -3- methoxy-4-oxo-3,3a,4,5,6,6a- hexahydropentalen-1-ylcarbamoyl]- 3-methylvaleric acid and benzyl (2*S*,3*S*)-2-[(3*RS*,3a*SR*,6a*RS*)-3-methoxy-4-oxo-3,3a,4, 5,6,6a-hexahydro pentalen-1-ylcarbamoyl]- 3-methylvalerate, have been synthesized. The synthetic compounds were highly phytotoxic on a range of weeds to show their potential application as herbicides [332].

Transformation of phytotoxin-resistant genes from the fungus into plants is another strategy. Aspterric acid (**259**) is a fungal phytotoxin. Aspterric acid-producing fungi have the self-resistance gene, *astD*, which was validated to be insensitive to aspterric acid (**259**). The fungal self-resistance gene *astD* has been deployed as a transgene in the establishment of plants to create aspterric acid-resistant crops. Aspterric acid (**259**) should be a promising lead for development as a broad-spectrum commercial herbicide [189].

The current review describes the phytotoxic activities of secondary metabolites from fungi. In fact, many fungal phytotoxins have other biological activities in addition to their phytotoxic activity. For example, emodin (**86**) exhibits antioxidant [342], antitumor [343], phytotoxic [86], insecticidal [344], antimicrobial [344], and acetylcholinesterase (AChE) and glutathione *S*-transferase (GST) inhibitory [344] activities. In addition, many other isolated fungal metabolites have not been evaluated for their phytotoxic activities due to either the lack of phytotoxic assays or not enough of the compounds could be isolated to perform toxicity assays. These metabolites remain to be further tested for their phytotoxicities. For example, herbarin (**47**) is a naphthoquinone congener. It was previously isolated from a few fungal species such as *Anteaglonium* sp. FL0768 [345] and *Corynespora* sp. BA-10763 [346], and later showed obviously phytotoxic activity [59].

In addition to their potential as herbicides, fungal phytotoxins have other potential applications in agriculture [1]. As different fungal species produce specific phytotoxins, this characteristic could be used to develop rapid, simple and specific methods to recognize plant diseases such as the development of practical kits (i.e., rapid test strip) to be used directly in the field by farmers. Furthermore, phytotoxins could be used to select plant varieties resistant to disease instead of using whole plant-pathogen systems. In this way, the disease resistance breeding can be accelerated.

## Data Availability

Not applicable.
